# NADP(H) allosterically regulates the interaction between ferredoxin and ferredoxin‐NADP^+^ reductase

**DOI:** 10.1002/2211-5463.12752

**Published:** 2019-11-15

**Authors:** Yoko Kimata‐Ariga, Yutaro Chikuma, Takashi Saitoh, Masayuki Miyata, Yuetsu Yanagihara, Kazukiyo Yamane, Toshiharu Hase

**Affiliations:** ^1^ Department of Biological Chemistry College of Agriculture Graduate School of Sciences and Technology for Innovation Yamaguchi University Yoshida Japan; ^2^ Laboratory of Regulation of Biological Reactions Division of Protein Chemistry Institute for Protein Research Osaka University Suita Japan; ^3^ Division of Pharmaceutics Hokkaido Pharmaceutical University School of Pharmacy Sapporo Japan; ^4^ Present address: Medical Corporation Yuseikai Osaka Japan

**Keywords:** allosteric regulation, ferredoxin, ferredoxin‐NADP^+^ reductase, negative cooperativity

## Abstract

Ferredoxin‐NADP^+^ reductase (FNR) in plants receives electrons from ferredoxin (Fd) at the end of the photosynthetic electron transfer chain and converts NADP^+^ to NADPH. The interaction between Fd and FNR in plants was previously shown to be attenuated by NADP(H). Here, we investigated the molecular mechanism of this phenomenon using maize FNR and Fd, as the three‐dimensional structure of this complex is available. NADPH, NADP^+^, and 2′5′‐ADP differentially affected the interaction, as revealed through kinetic and physical binding analyses. Site‐directed mutations of FNR which change the affinity for NADPH altered the affinity for Fd in the opposite direction to that for NADPH. We propose that the binding of NADP(H) causes a conformational change of FNR which is transferred to the Fd‐binding region through different domains of FNR, resulting in allosteric changes in the affinity for Fd.

## Abstract 2

The interaction between ferredoxin (Fd) and Fd‐NADP^+^ reductase (FNR) in plants is attenuated by NADP(H). Site‐directed mutations of FNR which change the affinity for NADPH altered the affinity for Fd in the opposite direction. We propose that the binding of NADP(H) leads to conformational changes of FNR, resulting in allosteric changes in the affinity for Fd.

## Graphical abstract

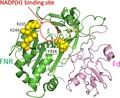

**Abbreviations**
cyt *c*: cytochrome *c*
Fd: ferredoxin
FNR: ferredoxin‐NADP^+^ reductase
ITC: isothermal titration calorimetry

Ferredoxin‐NADP^+^ reductase (FNR) in plants receives electrons from ferredoxin (Fd) at the end of the photosynthetic electron transfer chain and converts NADP^+^ to NADPH. FNR and Fd form a 1 : 1 complex to perform electron transfer, and the details of the protein–protein interaction were clarified by X‐ray crystallography in conjugation with other analyses 1, 2. Fd distributes electrons to various metabolic enzymes other than FNR in chloroplasts, and its role in regulating the electron distribution to these enzymes based on protein–protein interaction has been pointed out 3, 4, 5. In the course of investigating the regulatory mechanism performed by Fd described above, we found that the binding of FNR in the extract of plant chloroplasts to Fd was weakened by NADP^+^, a substrate of FNR, using Fd‐affinity chromatography (as shown in Fig. 1). Regarding this phenomenon, previous report of absorption spectrum analysis of purified spinach proteins 6 pointed out that the affinity between the FNR and Fd was decreased by NADP(H), which was considered to be a part of negative cooperativity to FNR by Fd and NADP(H) 6, reviewed in 7, 8. Measurements of dissociate constants between FNR and Fd by isothermal titration calorimetry (ITC) and tryptophan fluorescence analysis also supported this finding using pea and Anabaena FNRs 9, 10, 11, 12, and this phenomenon has been thought to play a role for driving the FNR reaction cycle efficiently.

**Figure 1:**
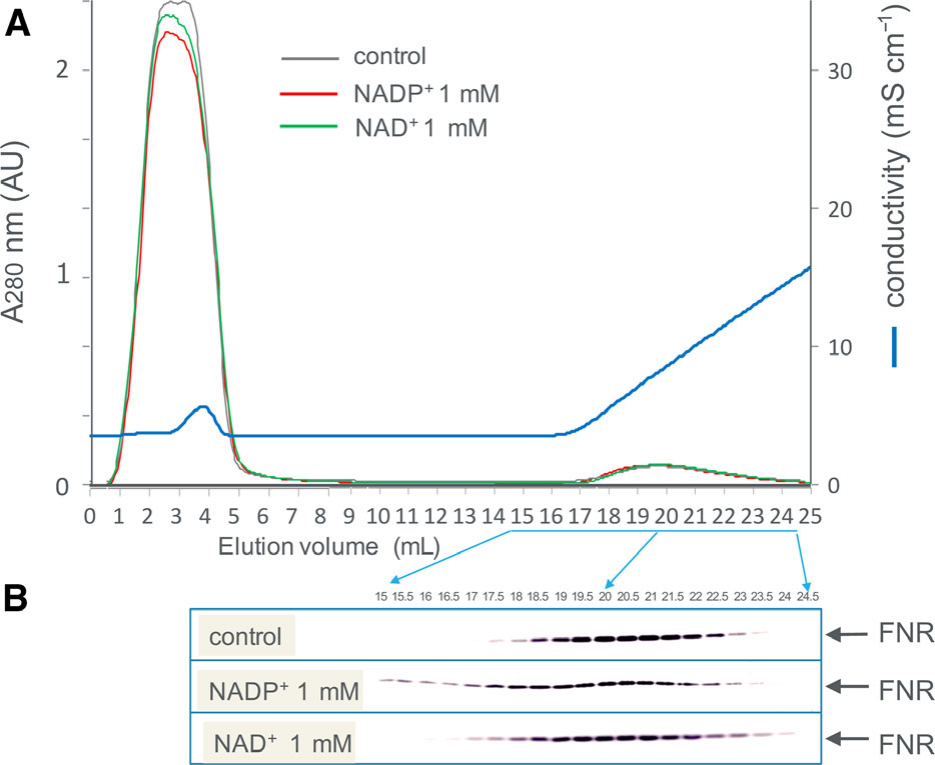
Fd‐affinity chromatography of the stroma fraction of spinach chloroplasts (A) and western blot analysis of the elution fractions using anti‐L‐FNR antibody (B). The chromatography was performed in the absence (control) and in the presence of NADP^+^ or NAD^+^ at 1 mm using maize Fd I‐immobilized resin (1 mL volume) developed with 0–300 mm NaCl in 50 mm Tris/HCl, pH 7.5. Fifteen microliters out of 500 μL of each twenty fractions (depicted in panel A and B) was loaded on the SDS/PAGE gel. Panel A shows the chromatography patterns in the absence (control) and in the presence of NADP^+^ and NAD^+^, monitored with absorbance at 280 nm.

In order to investigate the molecular mechanism of this phenomenon, here we studied the influence of NADP^+^ and NADPH on the binding between Fd and FNR by different methods (Fd‐affinity chromatography, and kinetic and calorimetric analyses) using maize recombinant proteins with known steric structures of FNR (accession code 1GAW) and Fd : FNR complex (accession code 1GAQ) 1, and the effects of site‐directed mutations at the sites involving the binding of NADP(H) on FNR were investigated. This is the first comprehensive study which investigates the effect of NADP(H) on the interaction between maize leaf Fd and FNR.

## Materials and methods

### Site‐directed mutagenesis of L‐FNR and preparation of recombinant proteins

Cloning and preparation of maize leaf FNR (L‐FNR) 13 and maize leaf Fd I 14, 15 were described previously. For the construction of L‐FNR mutants, the QuikChange Site‐Directed Mutagenesis Kit (Agilent Technologies, Santa Clara, CA, USA) was used according to the manufacturer’s instructions. The synthetic oligonucleotides used for the mutagenesis are shown in Table S1. The mutation sites and the sequence integrity of the entire coding region of L‐FNR were confirmed by DNA sequencing. Absorption spectra of the mutant proteins in the UV‐visible region were essentially the same as those of the parental FNR.

### Affinity chromatography using Fd‐immobilized resin

Isolation of the stroma fraction from spinach leaves was described previously 16. The stroma fraction derived from 500 μg on a chlorophyll basis of the chloroplast was used for each analysis. Small‐scale affinity chromatography using maize Fd I‐immobilized resin was performed as described previously 13. The gradient scale was varied depending on the experiments. The resulting fractions were analyzed by SDS/PAGE and/or western blotting using anti‐L‐FNR antibody as described previously 13.

### Enzymatic analysis

Enzyme activity of L‐FNR was measured using a grating microplate reader (model SH‐1000Lab; CORONA, Hitachinaka, Ibaraki prefecture, Japan). The activity of NADPH‐dependent electron transfer from FNR to Fd was measured using cytochrome *c* (cyt *c*) as a final electron acceptor basically as described previously 13 in the reaction mixtures containing different concentrations of NADPH. Diaphorase activity of FNR using DCPIP as an electron acceptor (two‐electron acceptor) was measured as described previously 13.

### Isothermal titration calorimetry

Calorimetric experiments were performed basically as described previously 17 except that NaCl was omitted in the reaction (50 mm Tris/HCl, pH 7.5). Protein samples were dialyzed against 50 mm Tris/HCl, pH 7.5, and degassed for 3 min before being loaded into the calorimeter. Calorimetric experiments were performed with an Auto‐iTC 200 instrument (GE Healthcare Biosciences, Chicago, IL, USA) at 298 K. In the injection syringe, 500 μm Fd I was titrated into 50 μm wild‐type or mutant L‐FNR in the ITC cell. Titration experiments consisted of 38 injections spaced at intervals of 150 s (filter period 5 s). The injection volume was 1.0 μL, and the cell was continuously stirred at 1000 r.p.m. Thermodynamic parameters of the complex formation between Fd and FNR were obtained as described previously 18 using the one set of independent binding site model supplied by the microcal origin 7.0 software, OriginLab Corporation, Northampton, MA, USA.

## Results and Discussion

### The effect of NADP(H) on the interaction between plant FNR and Fd

**Figure 2:**
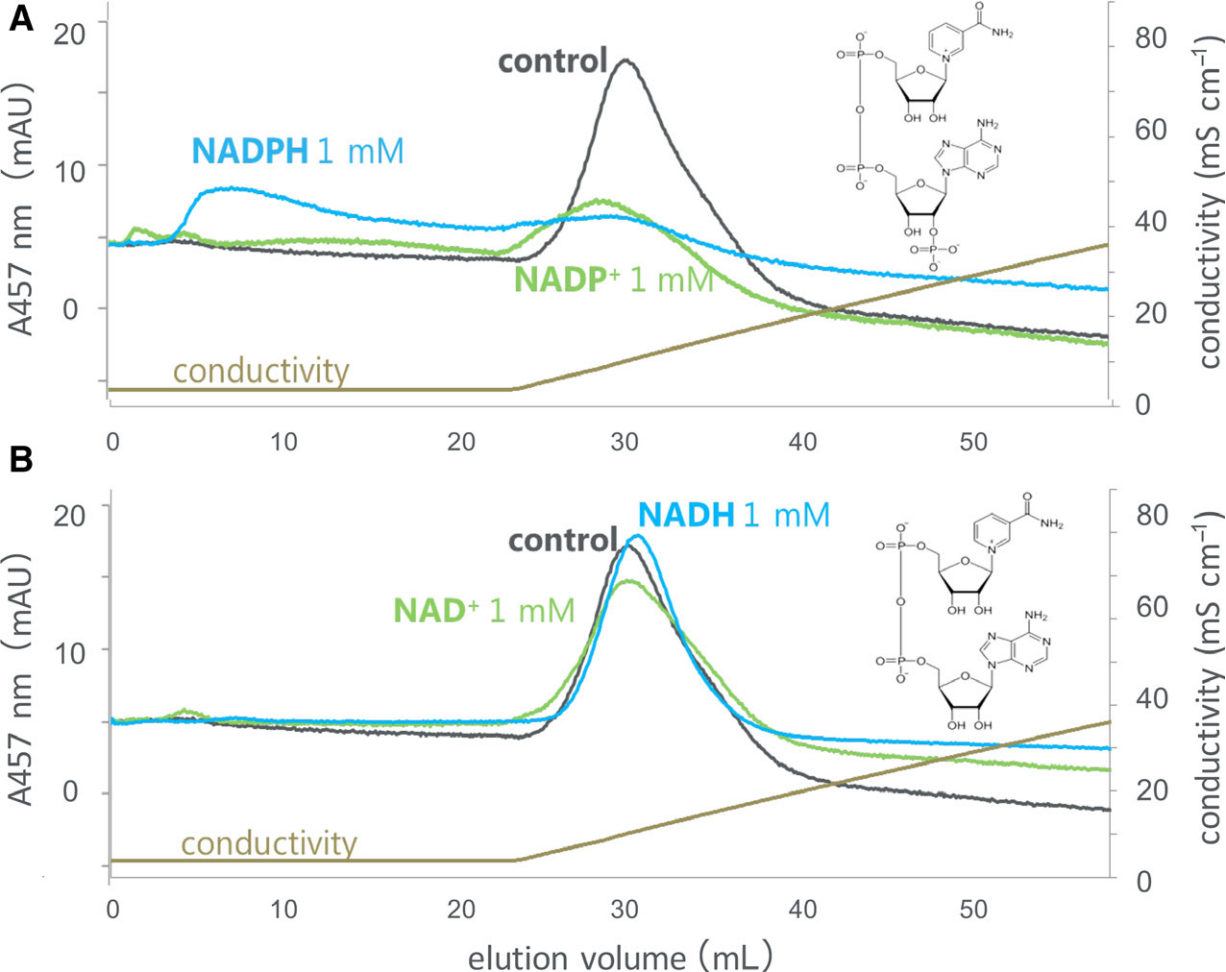
Fd‐affinity chromatography of recombinant maize L‐FNR in the absence (control) and in the presence of NADP^+^ (green) or NADPH (blue) at 1 mm (A) and NAD^+^ (green) or NADH (blue) at 1 mm (B). The chromatography was developed with 0–300 mm NaCl in 50 mm Tris/HCl, pH 7.5, and FNR was monitored at 457 nm. The structures depicted in panels (A) and (B) are NADP^+^ and NAD^+^, respectively. The reason for the apparent small peak area with NADP^+^ could be partly because (a) in the presence of NADP^+^, certain amount of FNR may have eluted before NaCl gradient applied; and (b) baseline could be slightly lower than other two. The reason for the differences in baselines is not clear at present.

**Figure 3:**
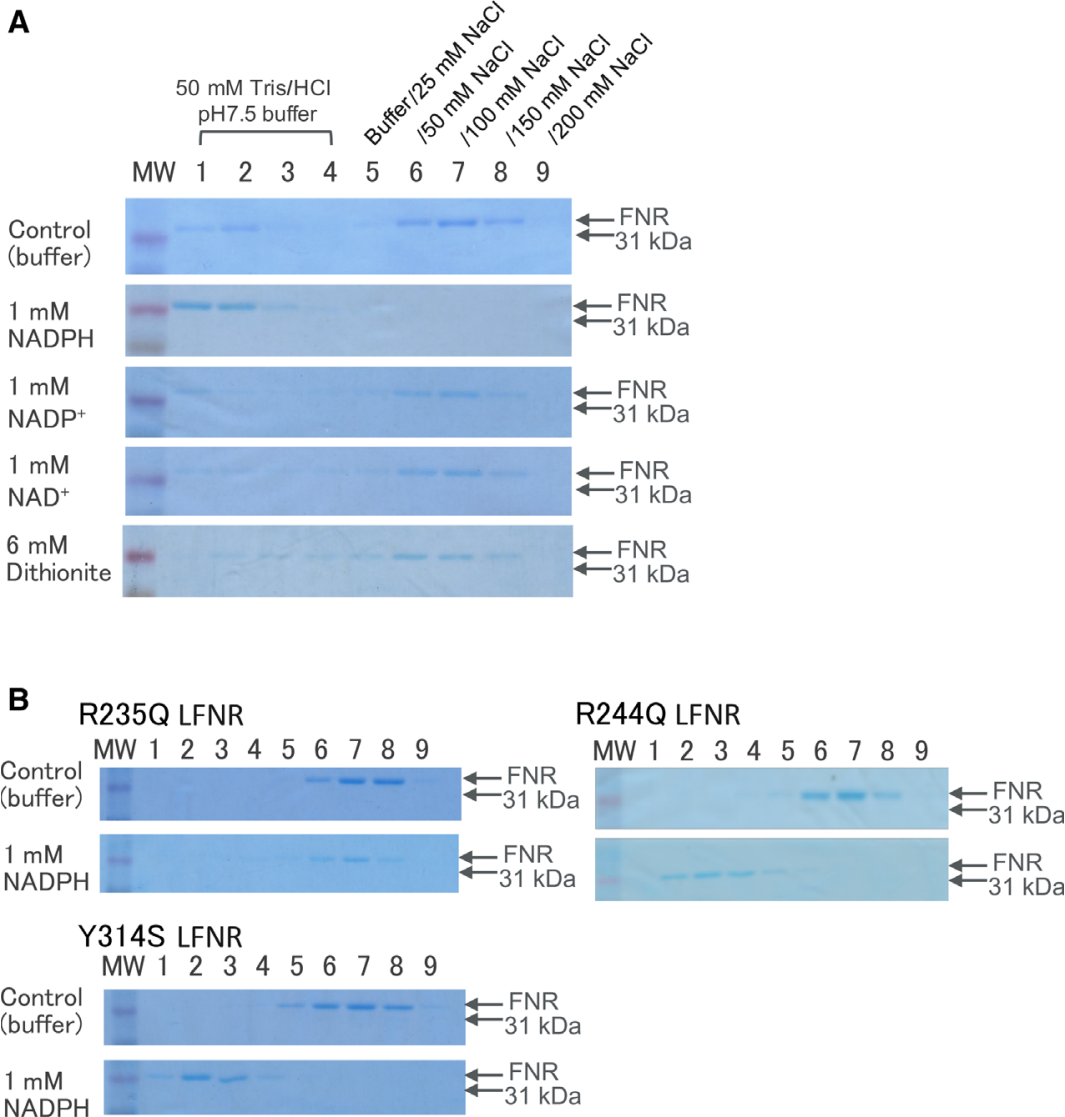
SDS/PAGE analysis of FNR in the fractions of Fd‐affinity chromatography. Recombinant maize L‐FNR (50 μL of 10 μm maize leaf FNR) was loaded on a small column (ca. 18 nmole of maize leaf Fd‐immobilized) and eluted with stepwise increasing concentrations of NaCl in the absence (control) and in the presence of NADPH, NADP^+^, or NAD^+^ at 1 mm or dithionite at 6 mm. The reason for the faint band observed in lanes 1 and 2, passed through the column under the conditions of control, NADP^+^, and NAD^+^, is not clear at present (A). Site‐directed mutants of maize L‐FNR were analyzed in the absence (control) and in the presence of NADPH at 1 mm (B). The numbering of the lanes 1–9 is the same as that of panel A. The addition of dithionite at 6 mm caused slight (0.16) changes in pH, but it did not significantly change the affinity of FNR for Fd, as observed at the bottom in panel A.

The interaction of Fd with FNR in the stroma fraction of maize chloroplasts was investigated by affinity chromatography using Fd‐immobilized resin and subsequent western blotting (Fig. 1). The FNR was bound to the Fd resin under the condition of 50 mm Tris/HCl, pH 7.5, and eluted during the linear gradient of 0–300 mm NaCl (control). In the presence of 1 mm NADP^+^, the FNR eluted slightly faster, while NAD^+^ at 1 mm only broadened the elution. In order to verify whether this effect is due to the property of FNR protein itself, recombinant maize leaf FNR, for which 3D structural information is available 1, was used for further analysis. Similar to the case of the chloroplast extract, the purified recombinant FNR, monitored with absorbance at 457 nm (absorbance peak of FNR), was bound to the Fd resin and eluted during the linear gradient of NaCl at around 60 mm (control in Fig. 2A), while in the presence of 1 mm NADP^+^, the FNR eluted slightly faster (green line in Fig. 2A). Again, NAD^+^ at 1 mm only broadened the peak (green line in Fig. 2B). This time, chromatography in the presence of 1 mm NADPH was also performed and showed that the large part of FNR was hardly bound to the Fd resin and eluted at the flow‐through fraction (blue line in Fig. 2A), while NADH at 1 mm did not cause such effect (blue line in Fig. 2B). The broad peaks observed in the presence of NADPH and NADP^+^ appear to reflect the heterologous interaction among Fd, FNR, and NADP(H) under these conditions. The presence of FNR protein was confirmed by SDS/PAGE in a similar analysis (Fig. 3A). Because this chromatography was performed under the aerobic condition, Fd and FNR proteins are mostly in the oxidized states except for the analysis under the condition of NADPH (1 mm), in which at least part of the Fd and FNR (< 100 μm in the resin) are thought to be in the reduced states. The addition of excess amount (6 mm) of dithionite during the chromatography considerably changed the color of Fd, indicating the reduction of Fd 19 (and probably FNR also), but hardly changed the elution pattern of FNR (Fig. 3A bottom). Whether the drastic change in the pattern in the presence of 1 mm NADPH was due to the effect of NADPH itself and/or was caused by the change in the redox state of the proteins is not clear at present.

**Figure 4:**
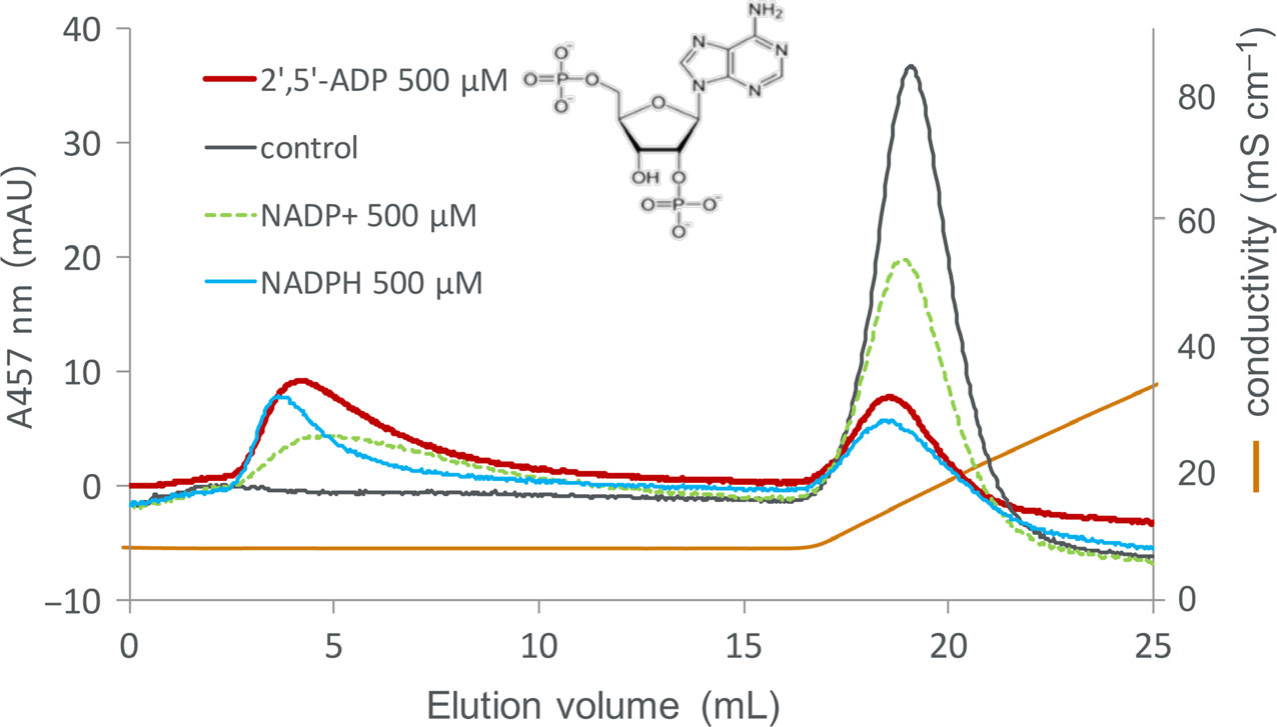
Fd‐affinity chromatography of recombinant maize L‐FNR in the absence (control) and in the presence of 2′,5′‐ADP (red), NADPH (blue), and NADP^+^ (green dot) at 500 μm. The chromatography was developed with 0–300 mm NaCl in 50 mm Tris/HCl, pH 7.5, and FNR was monitored at 457 nm. The structure of 2′,5′‐ADP is depicted in the figure.

In order to investigate which part of NADP(H) is responsible for the effect, Fd‐affinity chromatography of the FNR was performed in the presence of 2′5′‐ADP, a part of NADP(H) lacking the nicotinamide mononucleotide moiety, and a known inhibitor of FNR (as depicted in Fig. 4). The chromatography in the absence and in the presence of NADP^+^ and NADPH at the same concentration (500 μm) was also performed for comparison. In the presence of NADP^+^ and NADPH, the peaks observed at similar position with the main peak of the control appear relatively larger than those at the condition of 1 mm performed in Fig. 2. Therefore, these peaks appear to decrease in a way that depends on the concentration of NADP(H). 2′5′‐ADP caused the faster elution of FNR (intermediate effect of NADPH and NADP^+^); therefore, this portion containing 2′‐phosphoryl group appears to be responsible for reducing the affinity with Fd. The reason for the apparent larger effect than that of NADP^+^ is probably because 2′5′‐ADP binds to FNR tighter than NADP^+^ (sevenfold difference in *K*_d_) 20, as will be discussed later. In addition, *K*_d_ of FNR : NADPH complex was reported to be about 6% of the *K*_d_ of FNR : NADP^+^ complex 20, which may explain the reason why NADPH conferred larger effect than NADP^+^ as shown in Figs 2A and 4.

**Figure 5:**
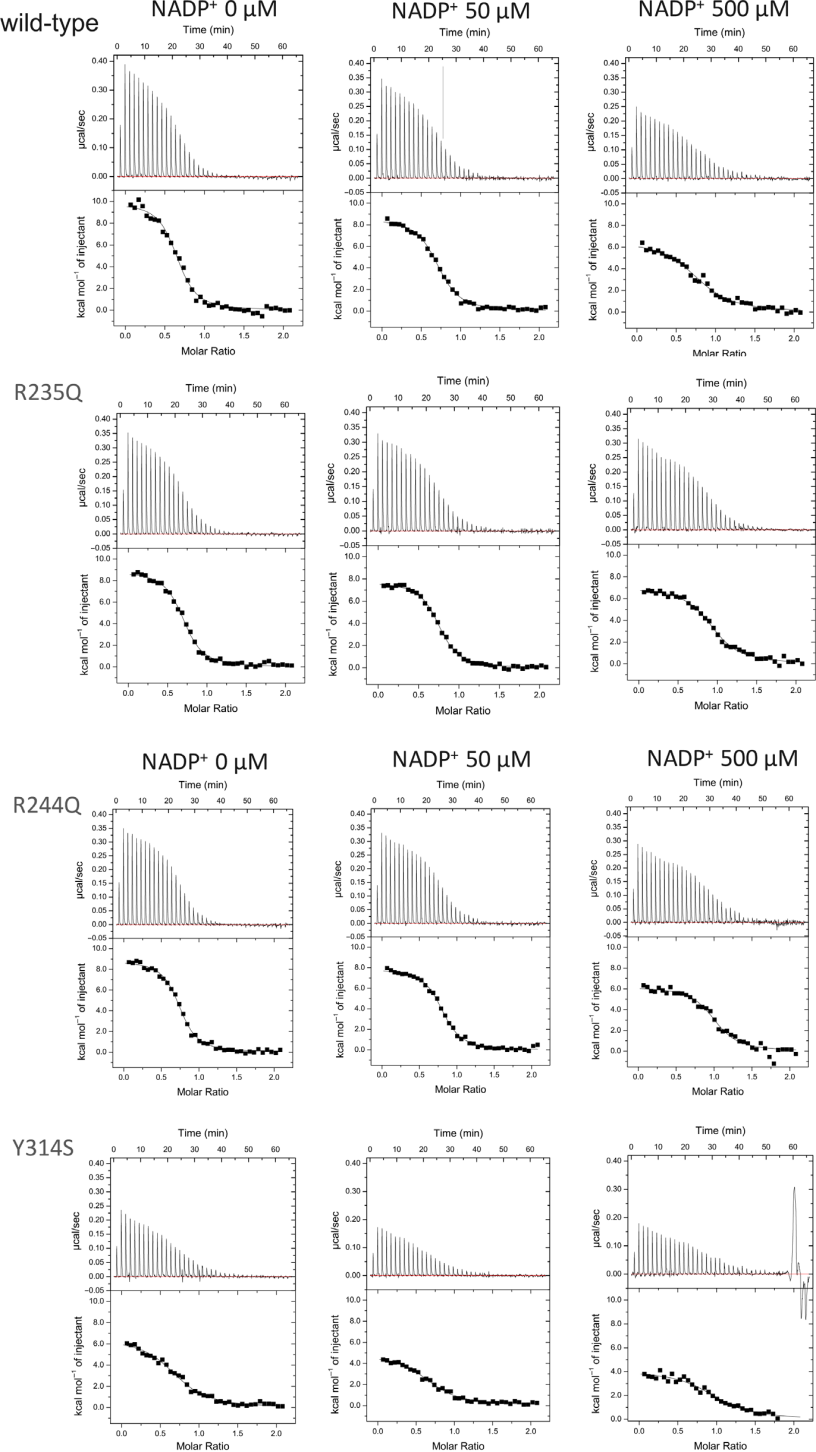
ITC thermograms of the titration of maize Fd to wild‐type, R235Q, R244Q, and Y314S L‐FNRs under different concentrations of NADP^+^ in 50 mm Tris/HCl pH 7.5 (upper panels). Normalized heat values plotted against the molar ratio ([Fd]/[FNR]; lower panels).

The effect of NADPH and NADP^+^ on the affinity between Fd and FNR was further investigated using different methods. Firstly, the effect of NADPH concentration on the affinity between Fd and FNR was investigated by kinetic analysis of NADPH‐dependent FNR‐Fd electron transfer reaction (Table 1, Fig. S2). By increasing NADPH concentration, the apparent *K*_m_ value for Fd remarkably increased from 3.5 ± 0.3 μm at 50 μm NADPH to 44 ± 10 μm at 1 mm NADPH (wild‐type in Table 1). Thus, the affinity between Fd and FNR was largely reduced by increasing NADPH concentration of physiological range (0.1–0.5 mm in the stroma of chloroplasts 21, 22). The *k*_cat_ value increased with the increase in the concentration of NADPH probably because NADPH would not reach the saturated concentration up to > 300 μm and possibly because the affinity for NADPH would become lowered in the presence of Fd (as compared to the *K*_m_ value of 46 μm in Table 3). The reason for the decrease in the *k*_cat_ at 1 mm NADPH is not clear at present. Next, the effect of NADP^+^ was investigated by the physical binding analysis using ITC (wild‐type in Table 2 and Fig. 5). The titration of Fd to wild‐type FNR showed a series of heat peaks indicating complex formation with heat uptake (Fig. 5), as reported previously 18. While the positive *∆H*_bind_ values display energetically unfavorable endothermic binding reaction, negative values of *∆G*_bind_ and −*T∆S*_bind_ (Table 2) indicate spontaneous Fd : FNR complex formation driven by entropy gain (positive *∆S*_bind_). These parameters of wild‐type FNR in the absence of NADP^+^ were very similar to those of our previous analysis under the same condition 18 and similar to other analyses of the same proteins under 25 mm sodium phosphate pH 6.0/50 mm sodium perchlorate at 300 K 23 and of Anabaena Fd and FNR under 50 mm Tris/HCl, pH 8.0 11. The resulting dissociation constant (*K*_d_) increased by addition of NADP^+^ into the titration reaction, although the extent of the increase was smaller than that of *K*_m_ value for Fd upon addition of NADPH (Table 1). The reason for the lower *n* values is not clear. But it could be due to the absence of salt in the reaction which would promote nonproductive interaction between Fd and FNR. In this respect, the addition of 500 μm NADP^+^ may have reduced such effect (increase in the *n*‐values other than Y314S). The effect of NADP^+^ addition on the values of *∆H*_bind_ (more favorable) and *∆S*_bind_ (less favorable) was similar to the previous ITC analyses using Anabaena proteins 10, 11.

**Table 1:** Steady‐state kinetic parameters of wild‐type and mutated maize L‐FNRs in the reactions of NADPH‐dependent cyt *c* reduction using Fd I at different NADPH concentrations. The values are mean ± SD of at least three independent measurements. ‘ND’ stands for ‘Not determined’.

FNR		NADPH conc			
		50 μm	250 μm	500 μm	1 mm
*K*_m_ for Fd (μm)	Wild‐type	3.5 ± 0.3	12 ± 3	22 ± 2.5	44 ± 10
	R235Q	0.30 ± 0.02	1.5 ± 0.3	2.1 ± 0.7	5.0 ± 0.7
	R244Q	0.75 ± 0.09	2.1 ± 0.2	3.7 ± 0.5	6.9 ± 1.4
	Y314S	17 ± 1	13 ± 3.0	18 ± 2	ND
*k*_cat_ (s^−1^)	Wild‐type	52 ± 7	97 ± 12	210 ± 20	150 ± 10
	R235Q	8.0 ± 1.3	32 ± 1	51 ± 6	53 ± 9
	R244Q	15 ± 1	49 ± 6	84 ± 8	70 ± 6
	Y314S	3.5 ± 0.4	3.6 ± 0.6	3.2 ± 0.5	ND

**Table 2:** Thermodynamic parameters of the complex formation between maize Fd and FNRs (wild‐type and mutated L‐FNRs) under different NADP^+^ concentrations, obtained by ITC (Fig. 5). The *n* values for the binding are shown in parentheses with *K*_d_.

FNR		NADP^+^ conc.		
		0 μm	50 μm	500 μm
*K*_d_ for Fd (μm)	Wild‐type	1.2 (0.7)	1.6 (0.7)	3.7 (0.8)
	R235Q	0.87 (0.7)	0.78 (0.7)	1.3 (0.9)
	R244Q	0.76 (0.7)	0.79 (0.8)	1.1 (1.0)
	Y314S	2.2 (0.7)	2.6 (0.7)	2.3 (0.7)
*ΔG*_bind_ (kcal·mol^−1^)	Wild‐type	−8.1	−7.9	−7.4
	R235Q	−8.3	−8.3	−8.1
	R244Q	−8.3	−8.3	−8.2
	Y314S	−7.7	−7.7	−7.7
*ΔH*_bind_ (kcal mol^−1^)	Wild‐type	9.8	8.8	6.7
	R235Q	8.3	7.3	6.3
	R244Q	8.3	7.4	5.8
	Y314S	5.6	4.3	3.6
−*TΔS *_bind_ (kcal·mol^−1^)	Wild‐type	−17.9	−16.7	−14.1
	R235Q	−16.5	−15.6	−14.5
	R244Q	−16.6	−15.7	−14.0
	Y314S	−13.3	−12.0	−11.3

In order to seek out the molecular mechanism of these effects, mutational analysis at the sites involving the binding of NADP(H) on the FNR was performed. Two types of site‐directed mutants, which would (a) reduce the electrostatic interaction with the phosphoryl group of NADP(H); and (b) modify the contact mode of the nicotinamide moiety of NADP(H), were prepared, and the effects of these mutations were analyzed.

### The effect of NADP(H) on the interaction between Fd and FNR mutants at NADP(H)‐binding sites, Arg235 and Arg244

X‐ray crystal structure of the complex between pea FNR mutant (Tyr308Ser) and NADP^+^ 24 revealed (a) the binding sites of adenosine ribose 2′‐phosphate (2′‐ phosphoryl group) of NADP^+^: Ser228, Arg229, Lys238, and Tyr240; and (b) the residue involving the binding of nicotinamide portion of NADP^+^, C‐terminal Tyr308, on the FNR. Among these residues, the side chains of Arg229 and Lys238 appear to contribute to the electrostatic interaction with negative charge of the phosphate. NMR chemical shift perturbation analysis of maize leaf FNR by additions of NADP^+^ and 2′5′‐ADP revealed the binding sites of NADP^+^ and 2′5′‐ADP on wild‐type maize leaf FNR 25, which are consistent with the binding sites of NADP^+^ obtained by the crystallographic study of the pea FNR mutant–NADP^+^ complex. Thus, site‐directed mutants of maize leaf FNR were prepared; Arg235 and Arg244 (depicted in Fig. S1B), corresponding to Arg229 and Lys238 of pea FNR, were substituted to Gln for the purpose of reducing the electrostatic interaction with the phosphoryl group of NADP(H). NADPH‐dependent diaphorase assay of the resulting mutants showed a large (7–11 times) decrease in the affinity for NADPH (R235Q and R244Q in Table 3) as expected. Then, whether the affinity for Fd would also change in these FNR mutants was examined using the methods described above. In the FNR‐Fd electron transfer reaction (Table 1), the *K*_m_ values for Fd were largely decreased (5–12 times) with these mutant FNRs at 50 μm NADPH and remained relatively low up to 1 mm NADPH as compared to those of wild‐type FNR, although the NADPH‐dependent increase in the *K*_m_ values was observed. The *k*_cat_ value was much smaller than the wild‐type FNR and increased with the increase in NADPH concentration because the affinity for NADPH was much lower than wild‐type FNR (Table 3). These results showed that the site‐directed mutants of FNR which decrease the electrostatic interaction with phosphoryl group of NADP^+^ exhibited the increased affinity for Fd, as compared to wild‐type enzyme. ITC analysis supports the results (Table 2 and Fig. 5); *K*_d_ values of these mutants with Fd were lower than wild‐type FNR in the absence of NADP^+^, and the increase in their *K*_d_ values by addition of NADP^+^ was smaller than that of wild‐type FNR. In comparison with wild‐type FNR, the complex formation of Fd and the mutant FNRs appeared to be favored mostly by smaller positive *∆H*_bind_. The precise mechanism for the changes in *∆H*_bind_ and *∆S*_bind_ on the mutation is not clear although the involvement of water dehydration upon the complex formation may be relevant 23, 26. Fd‐affinity chromatography of these mutant FNRs in the absence and in the presence of NADPH at 1 mm also supports the above results (Fig. 3B); the effect of NADPH on the elution profile was smaller with these mutants (slower elution), especially with R235Q, as compared to wild‐type FNR (1 mm NADPH in Fig. 3A).

**Table 3:** Steady‐state kinetic parameters of wild‐type and mutated maize L‐FNRs in the reactions of NADPH‐dependent diaphorase activity using DCPIP. The values are mean ± S.D. of at least three independent measurements.

FNR	*K*_m_ for NADPH (μm)	*k*_cat_ (s^−1^)
Wild‐type	46 ± 3	76 ± 14
R235Q	500 ± 120	60 ± 7
R244Q	310 ± 60	139 ± 13
Y314S	0.27 ± 0.08	41 ± 6

### The effect of NADP(H) on the interaction between Fd and FNR mutant at C‐terminal Tyr

Structural analysis of the complex between pea FNR (Tyr308Ser) mutant and NADP^+^ 24 indicates that the nicotinamide moiety of NADP^+^ occupies the equivalent position to the aromatic ring of the C‐terminal Tyr in the wild‐type FNR structure (depicted in Fig. S1A,B). Thus, NADP(H) binding on the wild‐type FNR has been thought to involve structural rearrangement in which the side chain of the Tyr or the C‐terminal backbone moves to make way for nicotinamide ring 7, 27, 28. Functional analysis of the pea FNR (Tyr308Ser) mutant 28 showed that the substitution of this Tyr by Ser allowed tighter binding to NADP(H) probably because of the lack of the competition with the nicotinamide ring 24. This also explains the reason why 2′5′‐ADP which lacks nicotinamide ring binds to FNR tighter than NADP^+^ 20. We prepared maize version of the equivalent FNR mutant (Tyr314Ser). NADPH‐dependent diaphorase assay of this mutant showed drastic (170 times) increase in the affinity for NADPH (Table 3), as expected from the results of the pea FNR mutant 28. Then, the affinity of this mutant for Fd was measured by the NADPH‐dependent electron transfer reaction (Table 1); the *K*_m_ value for Fd was higher than wild‐type at 50 μm NADPH (17 μm vs. 3.5 μm), but remained unchanged up to 500 μm NADPH. The *k*_cat_ values remained rather low (less than one fifteenth of wild‐type), while the diaphorase activity reached about half of wild‐type FNR (Table 3). The reason is not clear, but possibly due to the slow release of NADPH from this mutant FNR and also due to some effect on the electron transfer process to Fd. At 1 mm of NADPH, the reaction rate of this mutant FNR with increasing concentrations of Fd was largely deviated from the theoretical curve of Michaelis–Menten, and the kinetic parameters could not be determined (there were some deviations even at the lower concentrations of NADPH, as shown in Fig. S3); the reason is not clear at present. These results showed that the site‐directed mutant (Tyr314Ser) which increased the affinity for NADPH exhibited the decreased affinity for Fd and that NADPH‐dependent changes in its affinity for Fd were lost. ITC analysis (Table 2 and Fig. 5) supports the results; *K*_d_ value of this mutant with Fd was higher than that of wild‐type FNR in the absence of NADP^+^ (2.2 μm vs. 1.2 μm) and remained unchanged up to 500 μm NADP^+^. In comparison with wild‐type FNR, *∆H*_bind_ was significantly decreased but −*T∆S*_bind_ was significantly increased, indicating that the complex formation is energetically more favorable but entropically less favorable. Fd‐affinity chromatography of Tyr314Ser mutant FNR (Fig. 3B) showed that the affinity for Fd was slightly decreased in the absence of NADPH compared to wild‐type FNR (Fig. 3A), which is consistent with the result of ITC (Table 2). In the presence of 1 mm NADPH, slightly slower elution of this mutant FNR compared to wild‐type FNR indicates the moderately higher affinity for Fd under the condition at 1 mm NADPH.

**Figure 6:**
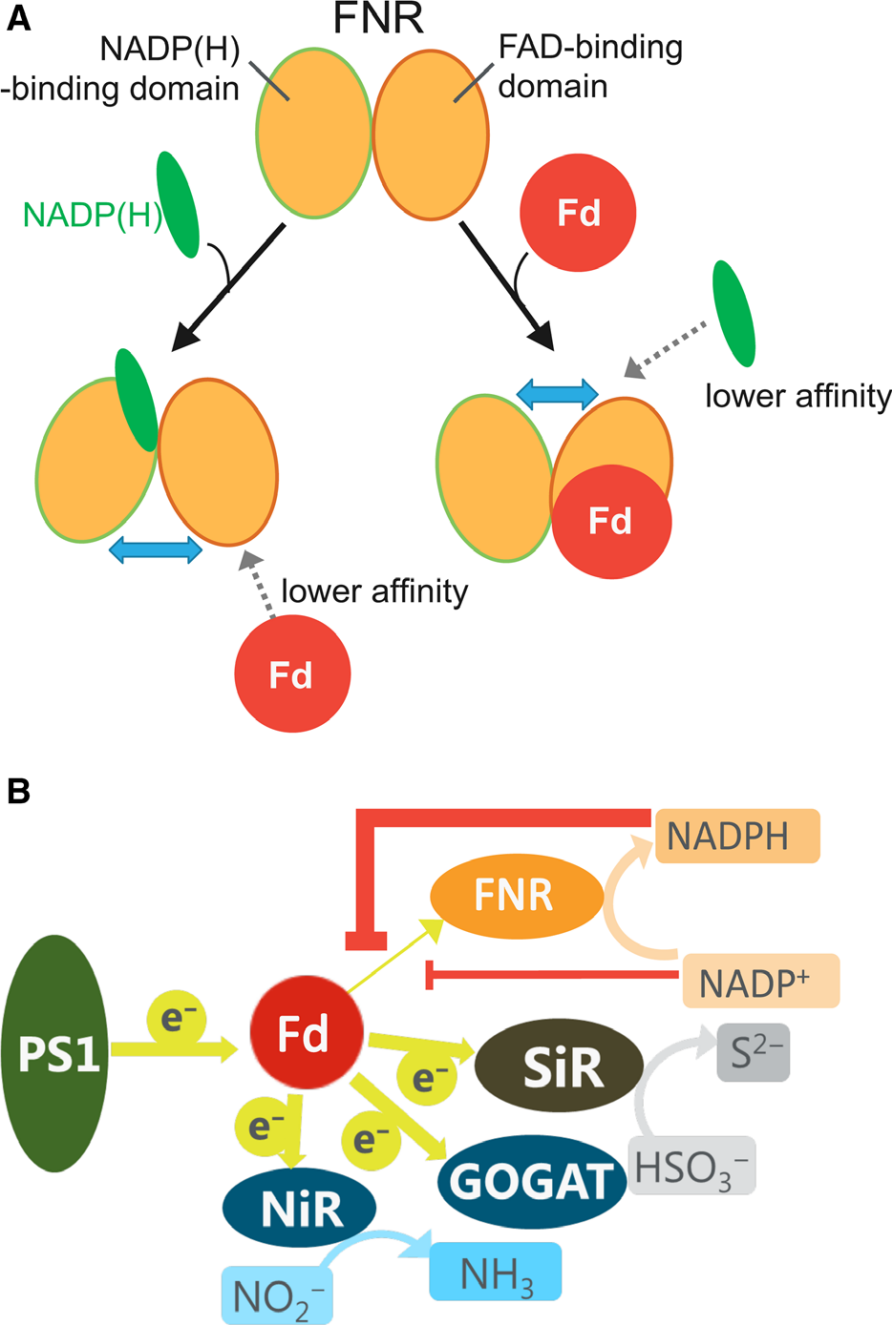
Schematic models for the reciprocal changes in the affinity of L‐FNR for Fd and NADP(H) (A) and for the changes in the electron distribution from Fd to Fd‐dependent enzymes in chloroplasts by the redox states of NADP(H) (B). In panel A, the binding of NADP(H) leads to the conformational (interdomain interaction) changes of FNR so that the affinity for Fd is lowered, and *vice versa*. In panel B, photosystem and Fd‐dependent enzymes are depicted; GOGAT: glutamate synthetase; NiR: nitrite reductase; PSI: photosystem I; SiR: sulfite reductase. Possible suppression of electron transfer from Fd to FNR by NADPH (thick orange line) and by NADP+ (thin orange line) is depicted.

These results of the two types of mutations showed that the FNR mutations which modify the affinity for NADPH lead to the changes in the affinity for the other ligand, Fd, in the opposite direction. This opposite effect does not seem to be due to the competition between the two ligands, but due to the allosteric effect, because the binding sites of these two ligands do not appear to overlap although fairly close. This allosteric effect is also supported by the following results: (a) Arg235 and Arg244 of FNR are far from Fd‐binding sites (Fig. S1B), but their mutations affect the affinity for Fd (Table 2); and (b) 2′5′‐ADP binds FNR at the region far from Fd‐binding sites 29, 30, but its binding to FNR reduces the affinity for Fd (Fig. 4). In this allosteric effect, the signal of NADP(H) binding on the FNR is thought to be transferred through FNR molecule to the Fd‐binding sites. This signal is thought to be transferred by way of conformational changes in the FNR protein, probably through the different domains of the FNR, from NADP(H)‐binding domain to the surface of Fd‐binding region which locates mostly in the FAD‐binding domain. Incidentally, the previous report of differential spectroscopy of spinach proteins 6 showed that the affinity of FNR for NADP^+^ was decreased by the addition of Fd, thus indicating negative cooperativity between the associations of Fd and NADP^+^. 3D structures of both the FNR‐Fd complex 1 and FNR(Tyr308Ser)‐NADP^+^ complex 24 are available, and the comparisons of the structures between the FNR in the free form and in these complexes show that the relative orientation of the two domains of the FNR is slightly altered upon the formation of both complexes (shown as arrows in Fig. S1). The NMR analyses of the addition of NADP^+^ or Fd to maize leaf FNR 23, 25 also showed that; (a) NADP^+^ binding caused the changes in the chemical shifts not only in NADP(H)‐binding domain, but also in FAD‐binding domain 25; and (b) Fd binding increased the flexibility in the NADP^+^‐binding domain 23. Crystal structure of FNR : NADP^+^ complex from *Anabaena* 31 revealed the structural rearrangements in the NADP^+^‐binding domain of FNR upon NADP^+^ binding. Therefore, either the binding of NADP^+^ and Fd to FNR appears to cause structural changes in a wide range of FNR molecule. These alterations in the relative orientation of the two domains of FNR may relate to the reciprocal changes in the affinity for the two ligands of FNR (as hypothesized in Fig. 6A). For example, upon the NADP(H) binding to FNR (Fig. 6A, Fig. S1A), the two domains appear to move to the way that the NADP(H)‐binding cavity is slightly closed and that the opposite Fd‐binding cavity may be slightly open. On the other hand, in the case of Fd binding (Fig. S1B), two domains appear to move in the opposite way as above so that the NADP(H)‐binding cavity could be slightly open (Fig. 6A). These motions may be the cause of the reciprocal changes in the affinity of FNR for Fd and NADP(H) (Fig. 6A). Currently, we are preparing the mutants which modify the interaction between the two domains of FNR to see how the affinity for the two ligands is modulated. There are other explanations for this phenomenon such that the increase in the surface negative charges caused by the binding of negatively charged Fd or NADP(H) reduces the affinity for the other ligand 27. However, Arg235Gln and Arg244Gln mutations, which reduce the positive charge on the surface of the FNR, lead to decrease in the affinity for NADPH (Table 3), but increase in the affinity for Fd (Table 2), and therefore, only the electrostatic influence does not seem to explain the phenomenon.

Regarding the physiological significance of this phenomenon, it has been pointed out that the binding of NADP(H) leads to dissociate the oxidized Fd bound to FNR in the reaction cycle of FNR, which contributes to drive the FNR reaction cycle 32, 33. Because the extent of the effects between NADP^+^ and NADPH was shown to be different in this study, physiological significance of the regulation of the affinity between Fd and FNR depending on the redox state in chloroplasts was considered. For example (as shown in Fig. 6B), as the NADPH/NADP^+^ ratio increases, the affinity between FNR and Fd may decrease, and thus, electron distribution from Fd to FNR may be suppressed. As a result, electron distribution from Fd to other Fd‐dependent proteins would be promoted and/or reorganized depending on the redox states in chloroplasts. These possibilities are now under investigation.

## Conclusions

The interaction between maize leaf FNR and Fd was shown to decrease with the addition of either NADP, NADP^+^, or 2′5′‐ADP, but to a different extent. FNR mutants which affected the affinity for NADPH altered the affinity for Fd in the opposite direction to that for NADPH. We propose that the binding of NADP(H) causes a conformational change of FNR, which is transferred to the Fd‐binding region through different domains of FNR, resulting in the changes in affinity for Fd allosterically.

## Conflict of interest

The authors declare no conflict of interest.

## Author contributions

TH, YC, and YK conceived the study and designed experiments; YC, TS, MM, YY, KY, and YK performed experiments; all authors analyzed the data; YK wrote the manuscript; YK, YC, TS, and TH made manuscript revisions.

## Supporting information


**Fig. S1.** Overlay of the crystal structures of pea leaf FNR (orange, code ID: 1QG0) and its Y308S FNR complex with NADP+ (green, code ID: 1QFZ) (A) and maize L‐FNR (orange, code ID: 1GAQ) and its complex with Fd (green, code ID: 1GAW) (B). FAD is shown as spheres, and NADP+ is shown as blue stick model. The [2Fe–2S] cluster of Fd (depicted with pink line) is shown as spheres. The arrows are explained in text. Mutation sites introduced in this study are shown as blown stick model in B.

**Fig S2.** Kinetics of cyt c reduction by wild‐type, mutant Y314, R235Q and R244Q FNRs using Fd at various NADPH concentrations.

**Fig S3.** Kinetics of diaphorase reaction of wild‐type, mutant Y314, R235Q and R244Q FNRs.

**Table S1.** Synthetic oligonucleotides used for the site‐directed mutagenesis.
