# Supplementary figures and images for: NADP(H) allosterically regulates the interaction between ferredoxin and ferredoxin‐NADP+ reductase
Source: FEBS Open Bio. 2019 Nov 15;9(12):2126–36. doi: 10.1002/2211-5463.12752 (PMC6886308; doi:10.1002/2211-5463.12752)

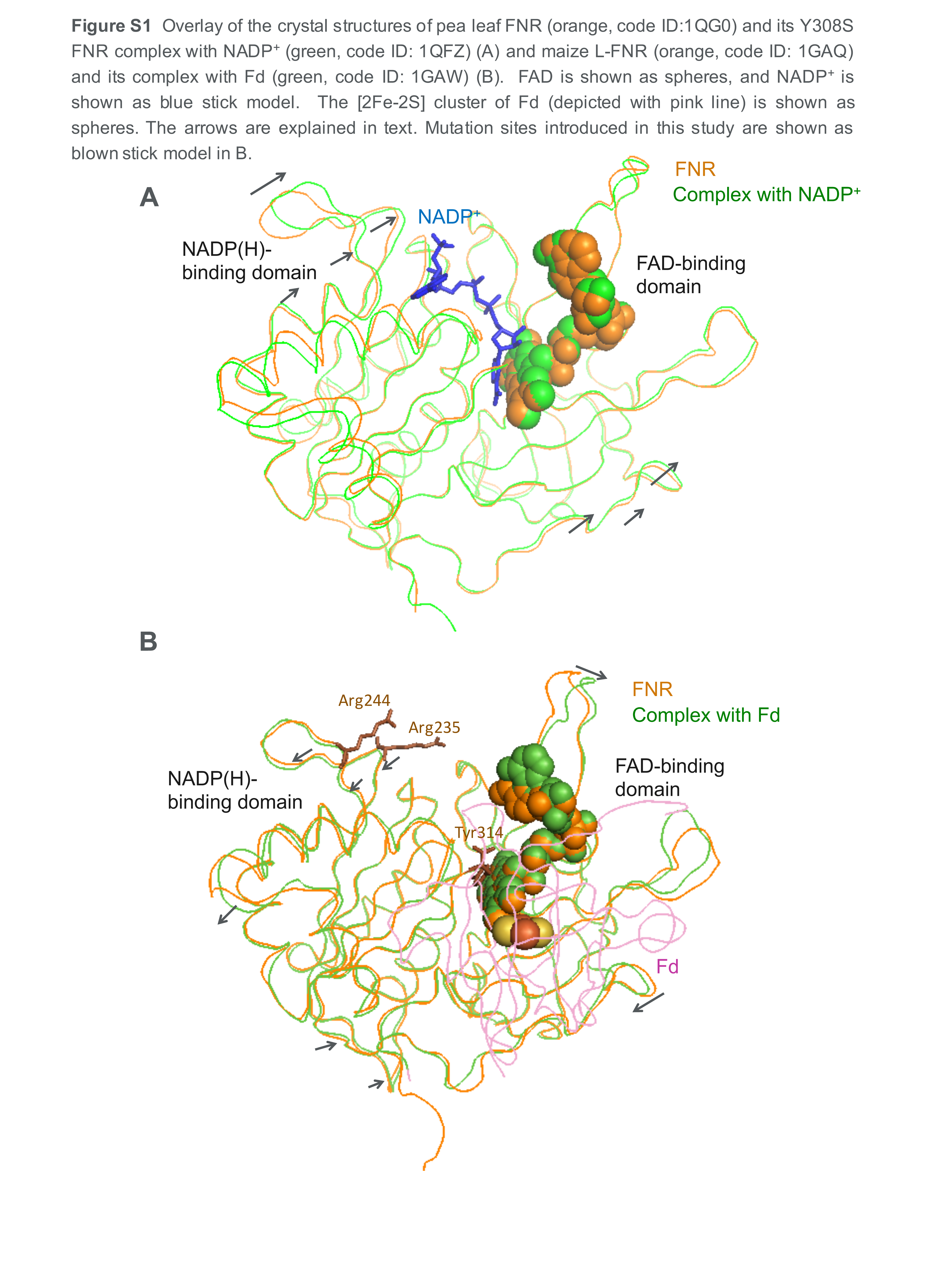

Supplement: Supplementary file 1 — Fig. S1. Overlay of the crystal structures of pea leaf FNR (orange, code ID: 1QG0) and its Y308S FNR complex with NADP+ (green, code ID: 1QFZ) (A) and maize L‐FNR (orange, code ID: 1GAQ) and its complex with Fd (green, code ID: 1GAW) (B). FAD is shown as spheres, and NADP+ is shown as blue stick model. The [2Fe–2S] cluster of Fd (depicted with pink line) is shown as spheres. The arrows are explained in text. Mutation sites introduced in this study are shown as blown stick model in B. [file FEB4-9-2126-s001.tif]
