# Supplementary material for: NADP(H) allosterically regulates the interaction between ferredoxin and ferredoxin‐NADP+ reductase
Source: FEBS Open Bio. 2019 Nov 15;9(12):2126–36. doi: 10.1002/2211-5463.12752 (PMC6886308; doi:10.1002/2211-5463.12752)
Supplement: Supplementary file 2 — Fig S2. Kinetics of cyt c reduction by wild‐type, mutant Y314, R235Q and R244Q FNRs using Fd at various NADPH concentrations. [file FEB4-9-2126-s002.pdf]

Cyt c reduction assay of wild-type FNR at 50 μM NADPH

numbers below X-axis: concentration of LFd (μM)

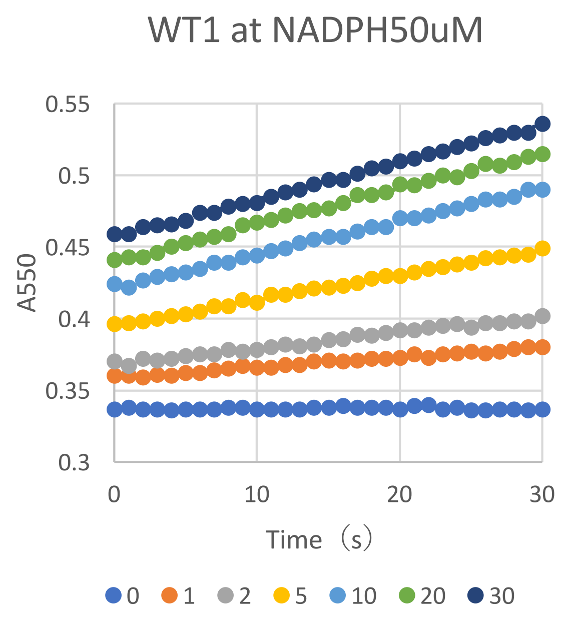

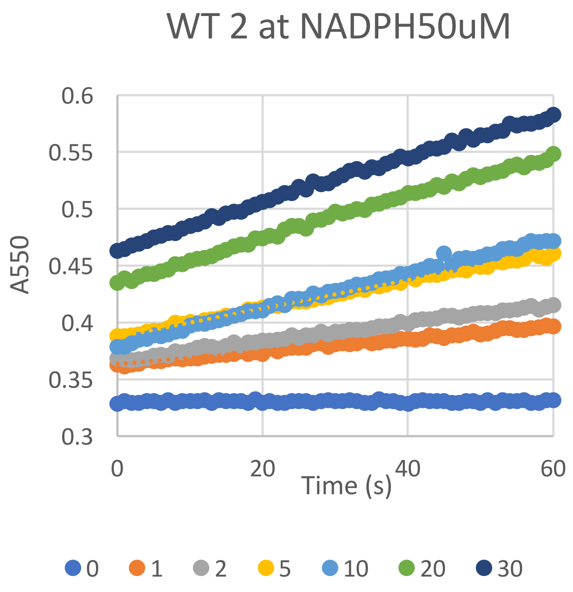

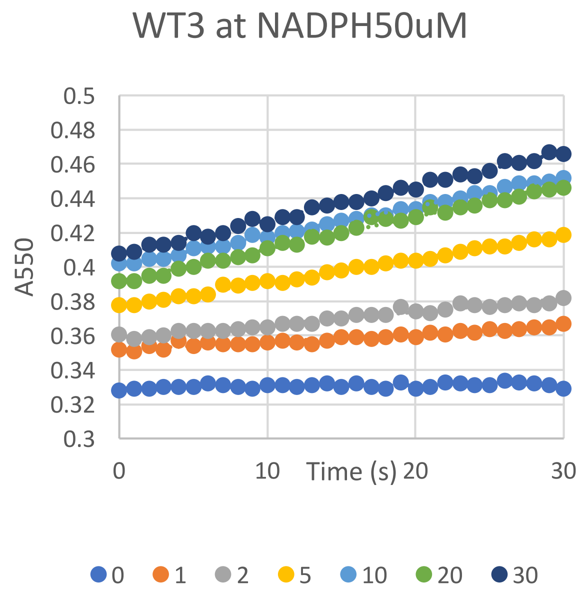

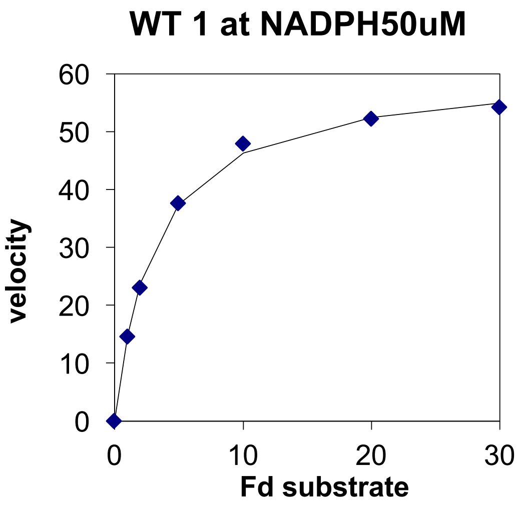

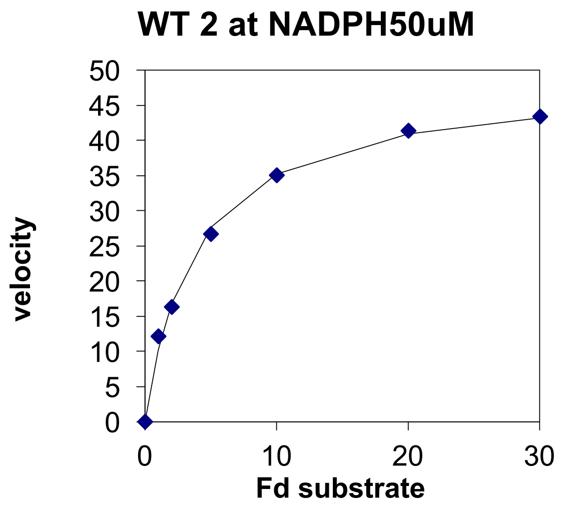

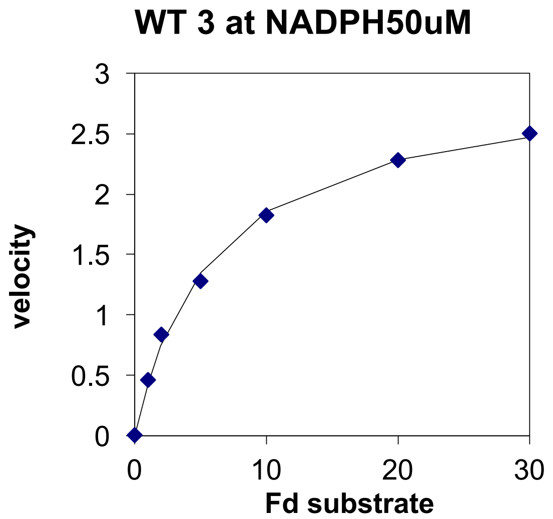

	1	2	3	Average	SD
Km (μM)=	3.106619487	3.808317081	3.699670092	3.53820222	0.308381564
Vmax (e/s)=	60.64829602	48.68677721	45.44797788	51.59435037	6.537221846

Y314S at 50 μM NADPH

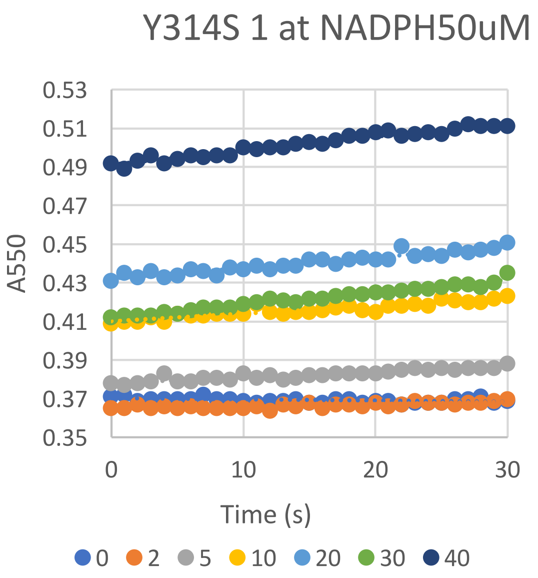

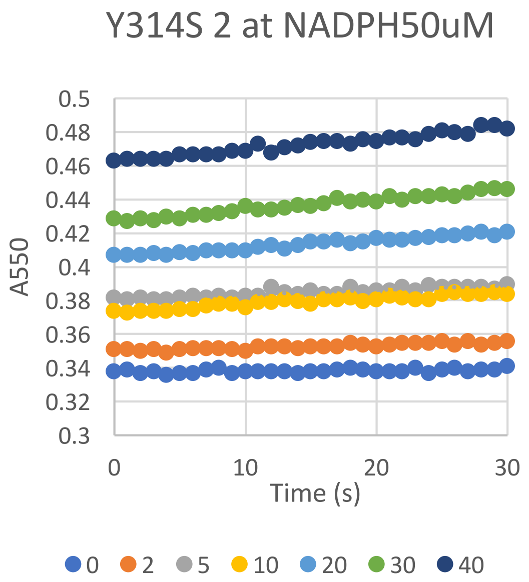

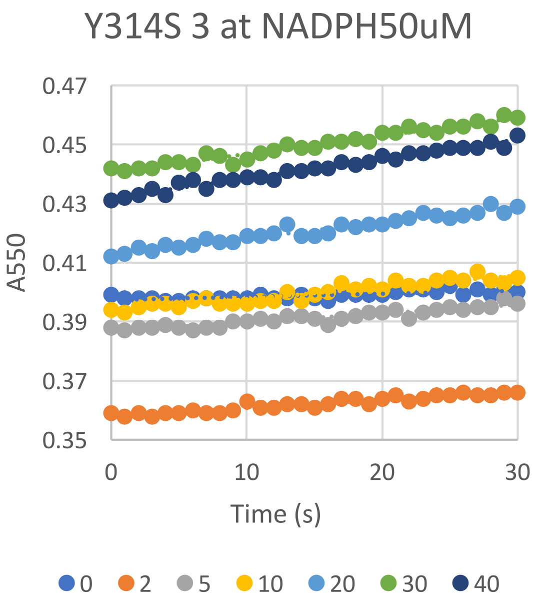

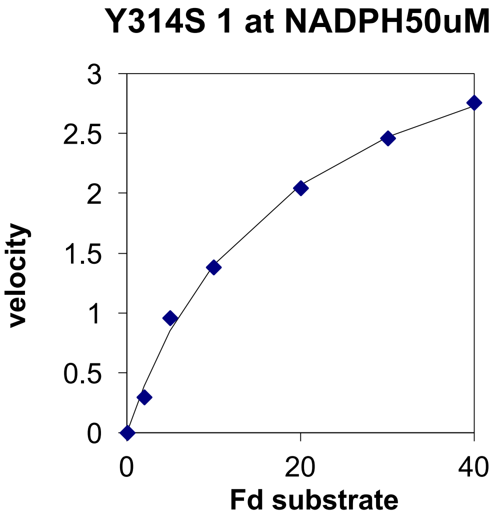

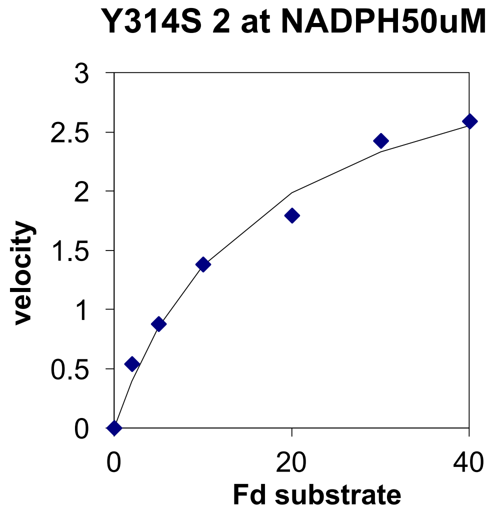

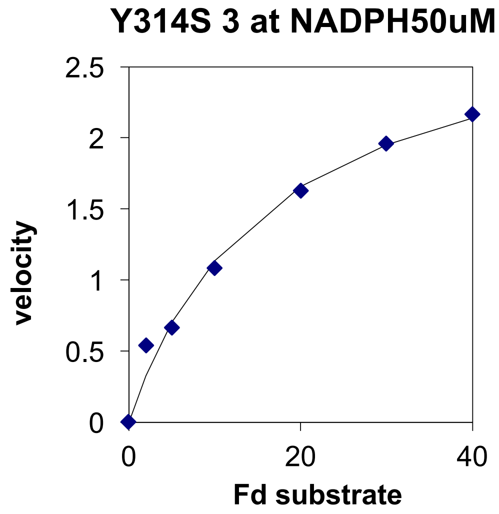

	1	2	3	Average	SD
Km (μM)=	18.57574261	16.05572332	16.4172826	17.01624951	1.112563203
Vmax (e/s)=	3.998349116	3.57623833	3.019211861	3.531266435	0.40099401

R235Q at 50 μM NADPH

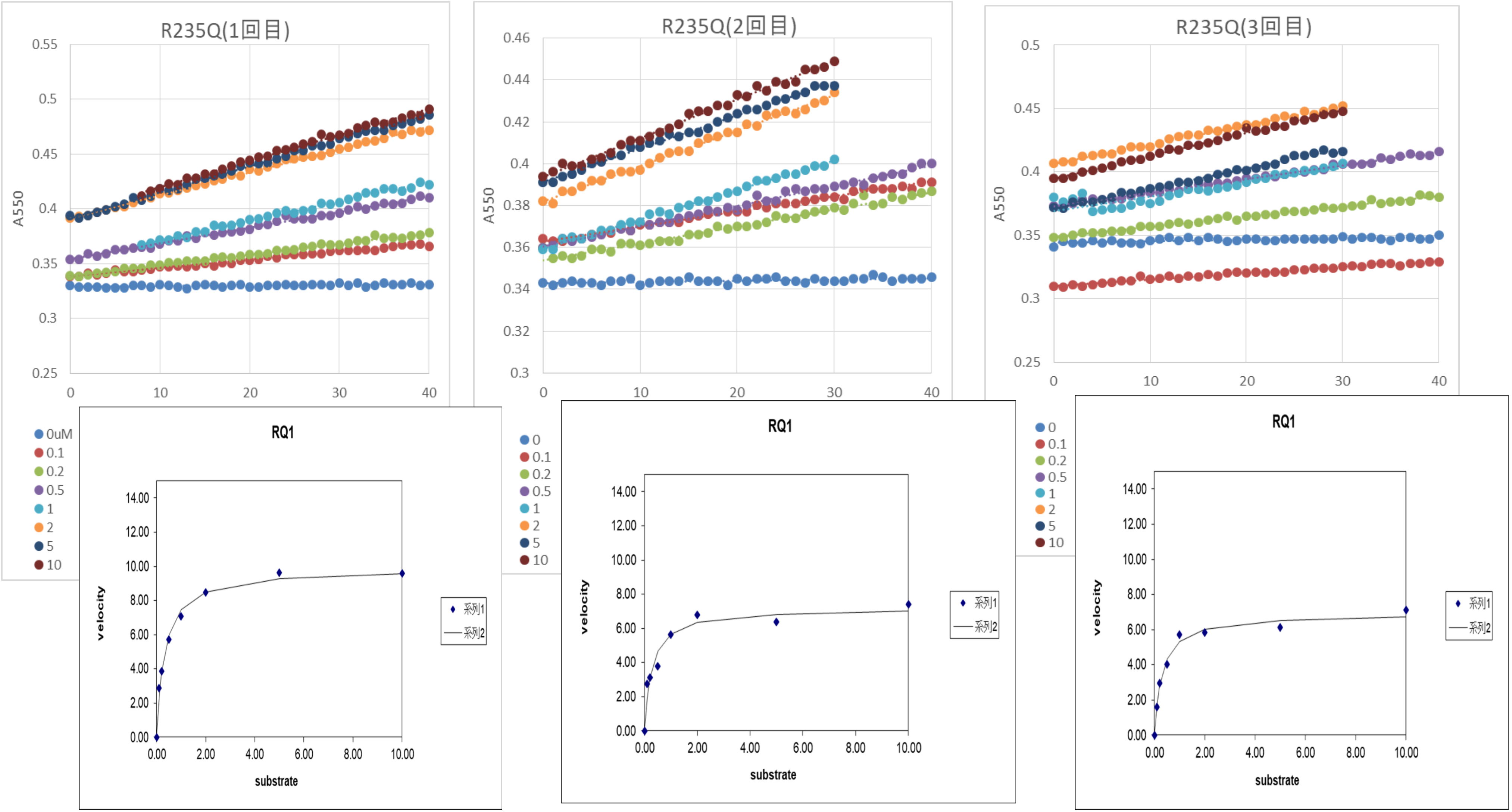

Km	Vmax
0.326	9.903

Km	Vmax
0.271	7.201

Km	Vmax
0.302	6.937

	Km (μM)		Vmax (e/s)	
R235Q	0.299±0.0225		8.014±1.34	

R244Q at 50 μM NADPH

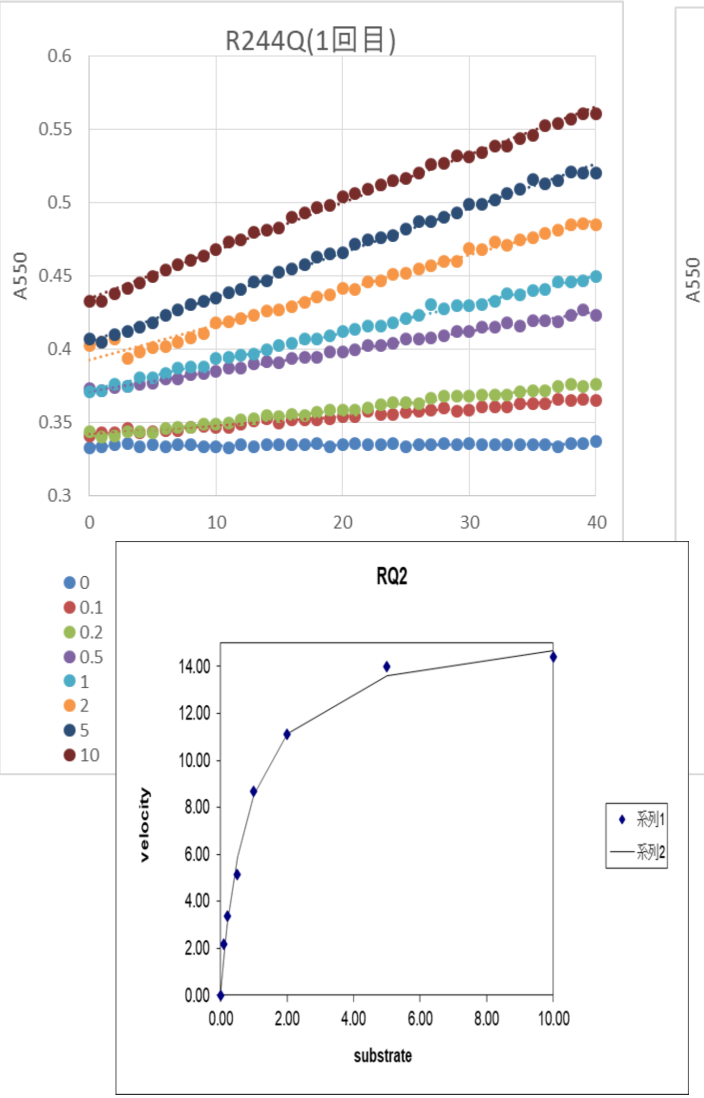

Km	Vmax
0.725	14.198

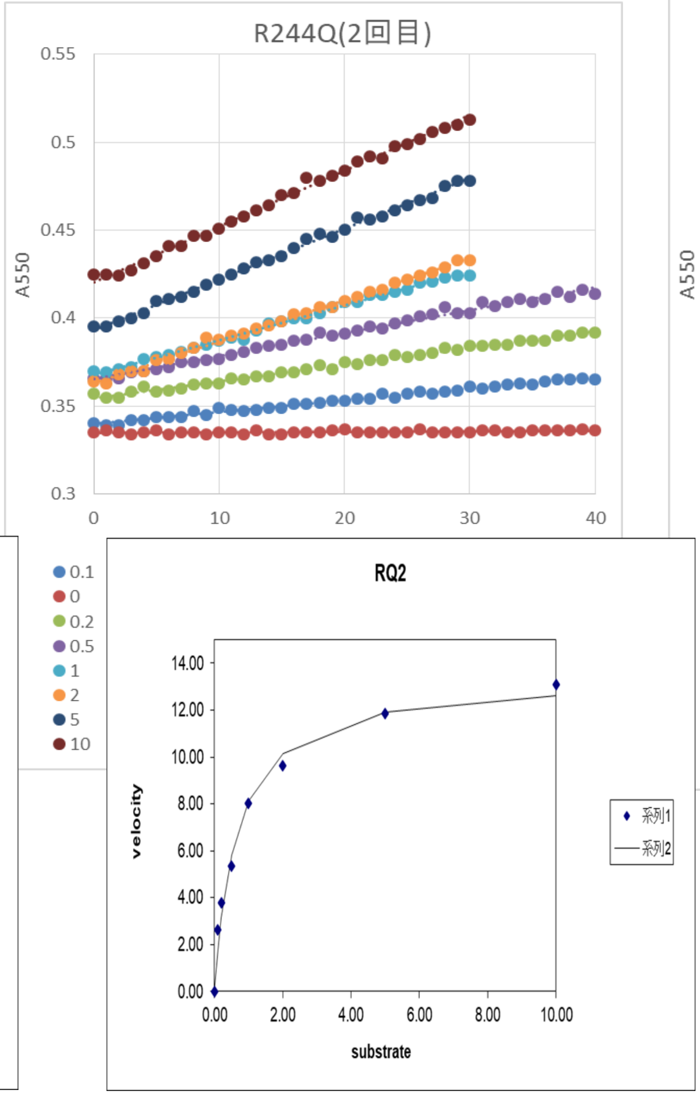

Km	Vmax
0.656	13.46

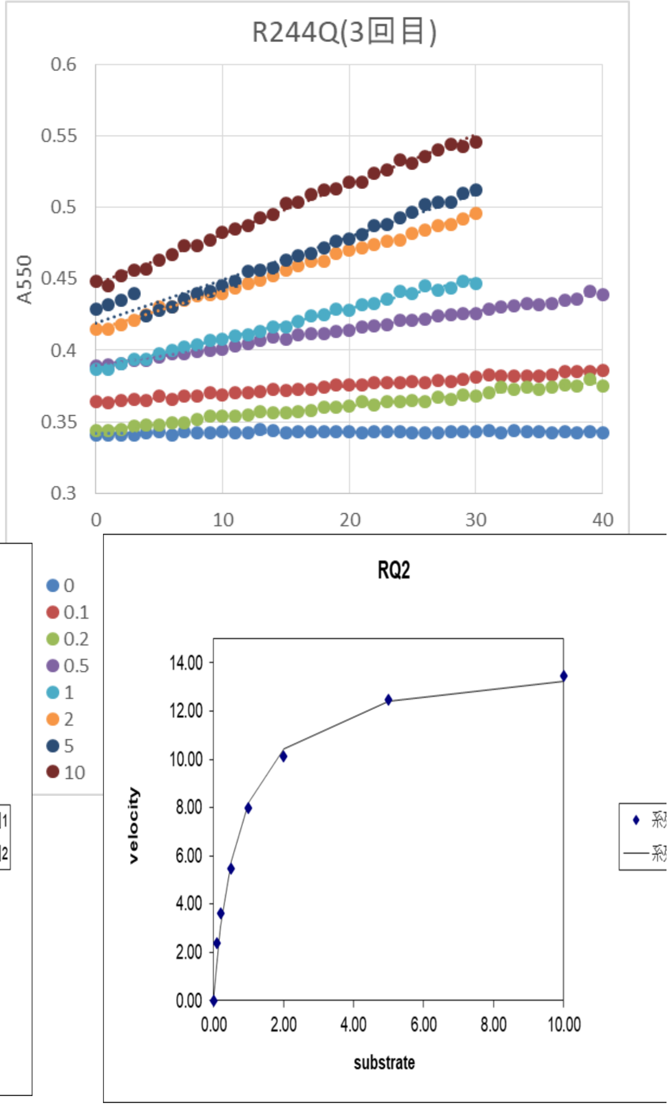

Km	Vmax
0.863	15.94

	Km (μM)	Vmax (e/s)
R244Q	0.748±0.086	14.53±1.039

Cyt c reduction assay of wild-type FNR at 250 μM NADPH

numbers below X-axis: concentration of LFd (μM)

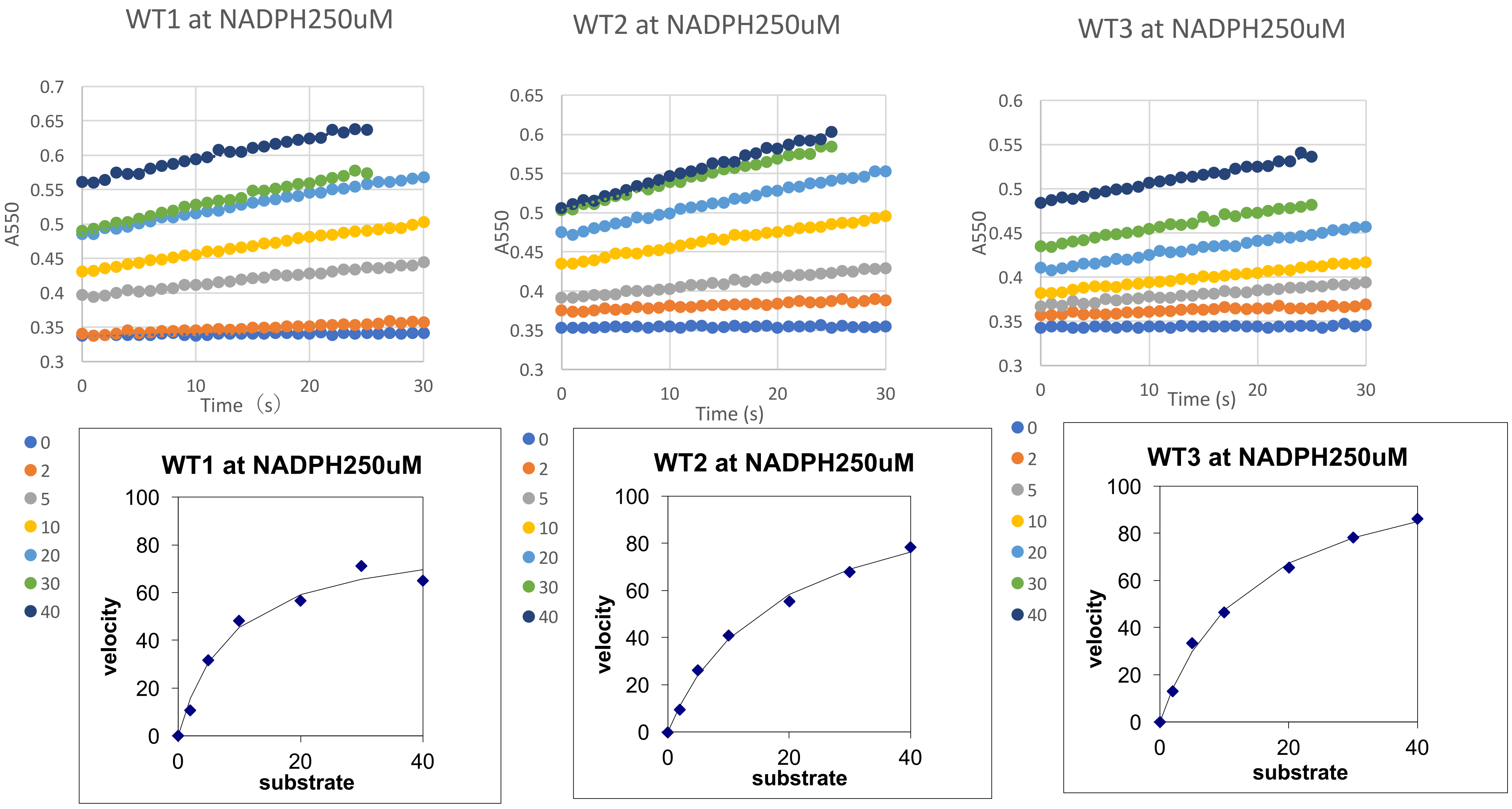

	1	2	3	4	5	6	Average	SD
Km (μM)=	8.689491789	17.93305898	14.22841464	9.493798154	11.22676394	10.17721815	11.95812428	3.19680672
Vmax (e/s)=	84.69469387	110.5180254	115.255227	89.03628878	88.56470355	94.82963176	97.14976172	11.59426389

cont. wild-type FNR at 250 μM NADPH

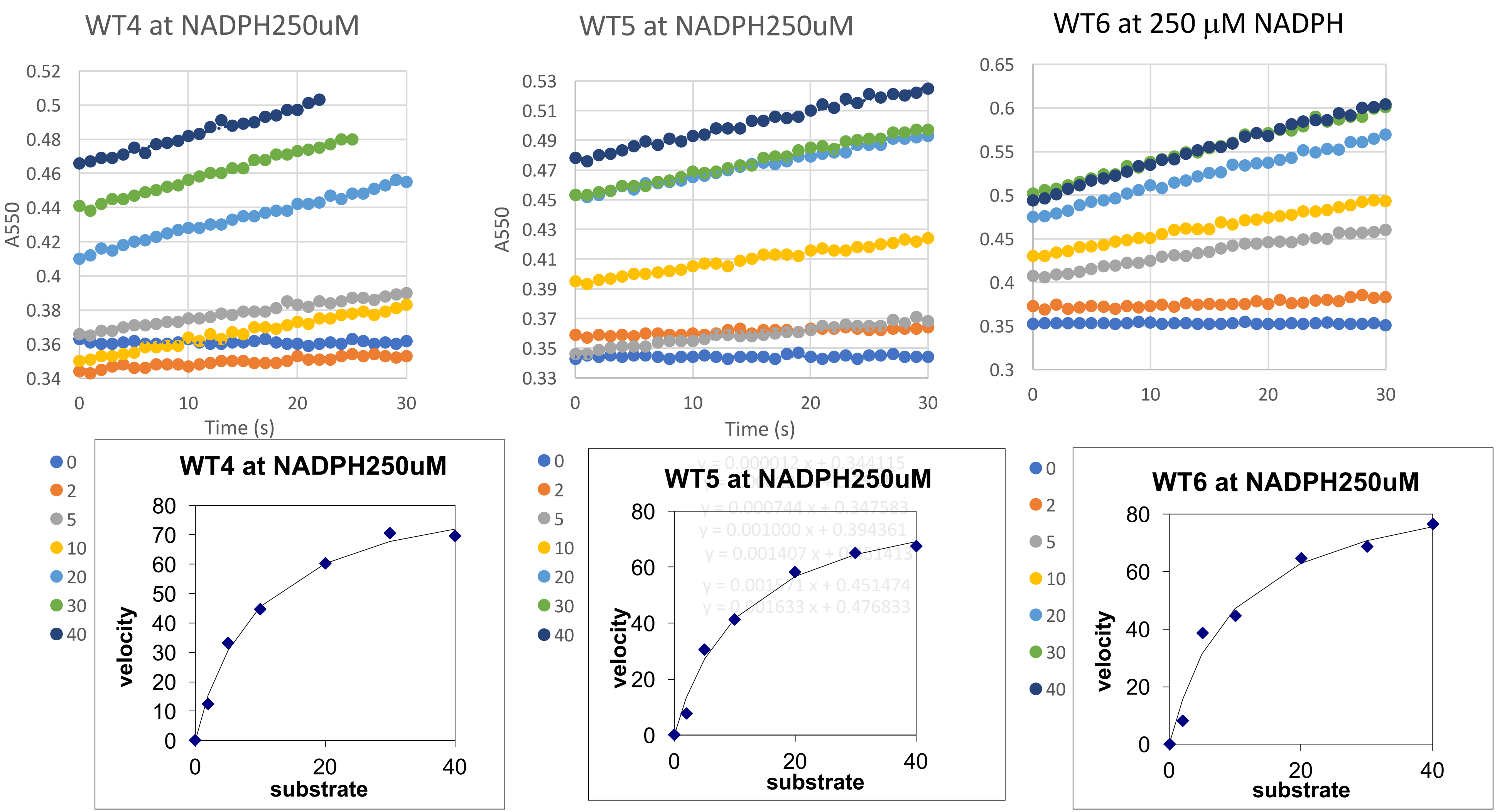

	1	2	3	4	5	6	Average	SD
Km (μM)=	8.689491789	17.93305898	14.22841464	9.493798154	11.22676394	10.17721815	11.95812428	3.19680672
Vmax (e/s)=	84.69469387	110.5180254	115.255227	89.03628878	88.56470355	94.82963176	97.14976172	11.59426389

Y314S at 250 uM NADPH

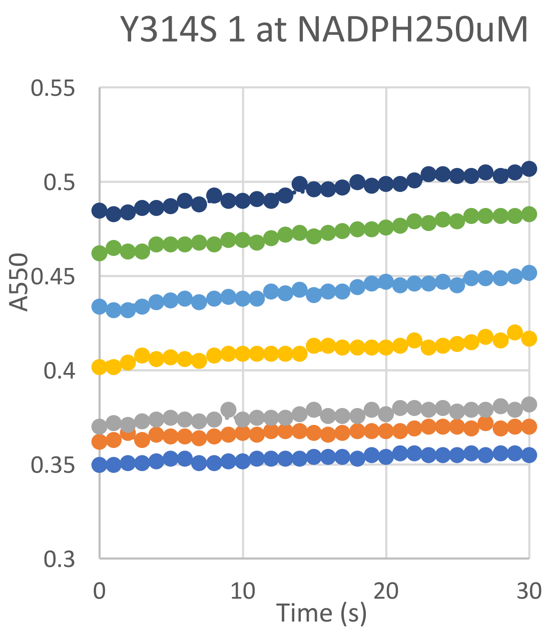

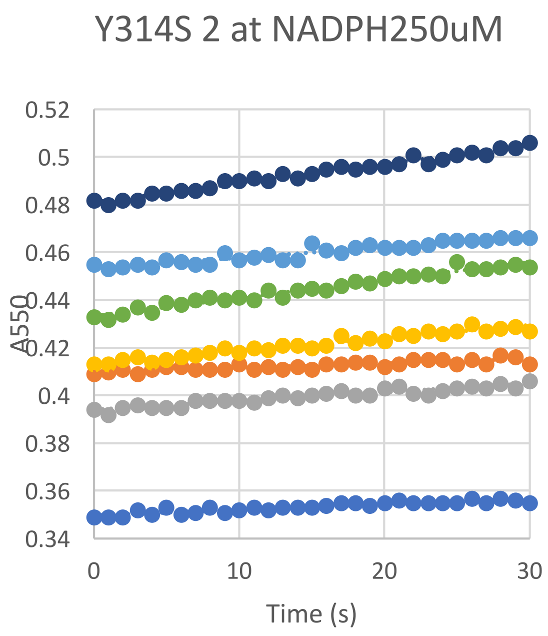

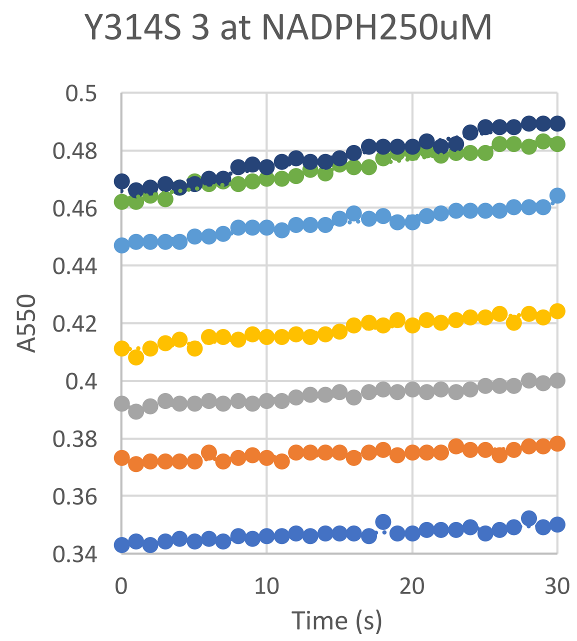

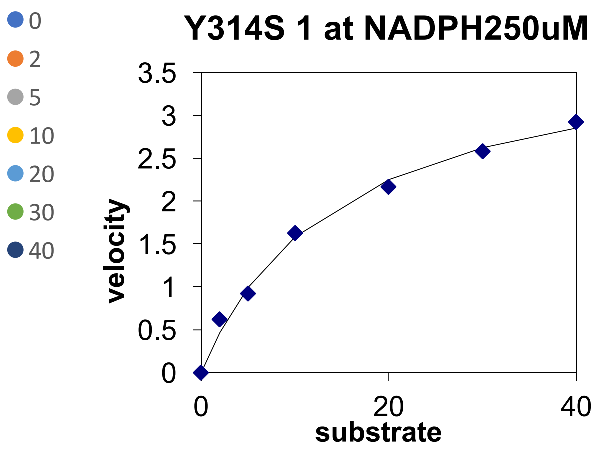

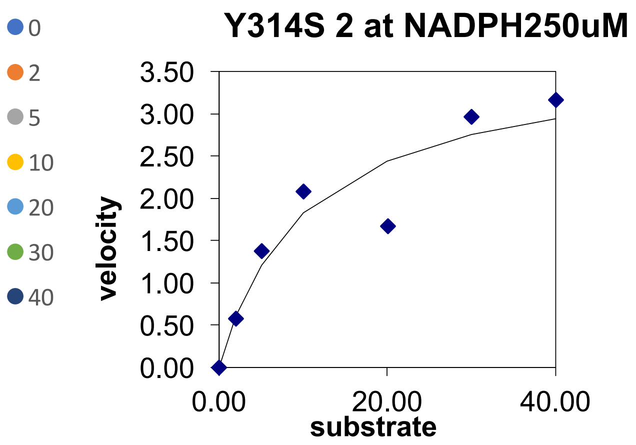

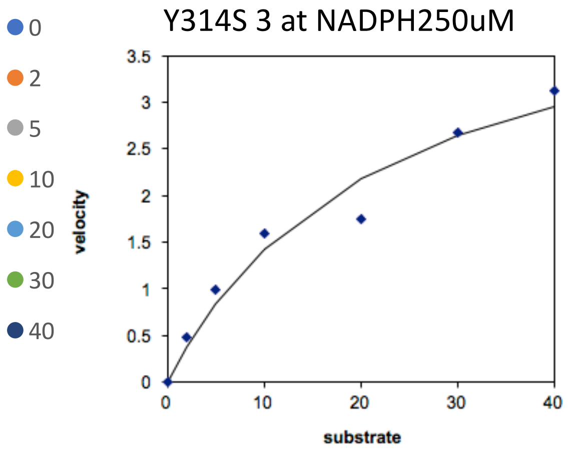

	1	2	3	4	5	Average	SD
Km (μM)=	14.62476831	9.402317583	17.56002178	14.13793672	10.16145187	13.17729925	3.019008771
Vmax (e/s)=	3.893403135	3.919003592	4.383900253	2.96273729	2.968351895	3.625479233	0.566424829

cont. Y314S at 250 uM NADPH

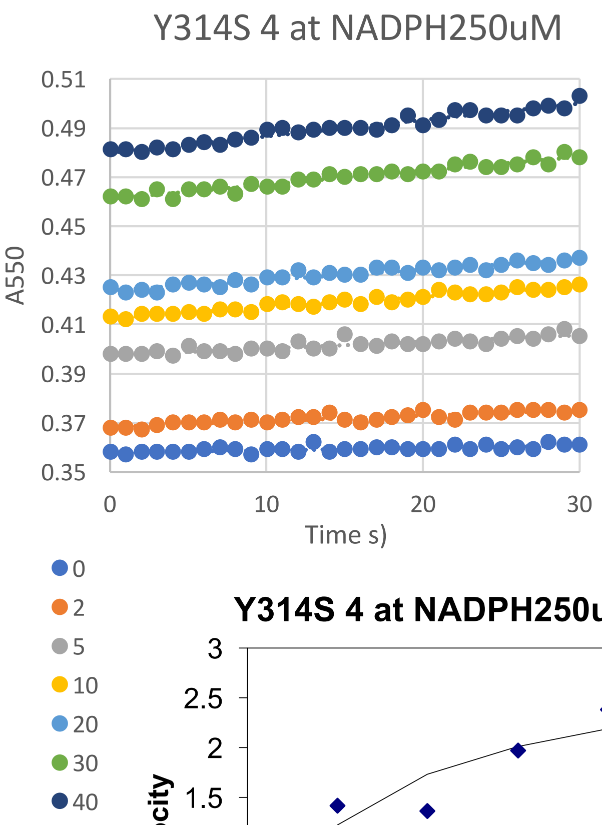

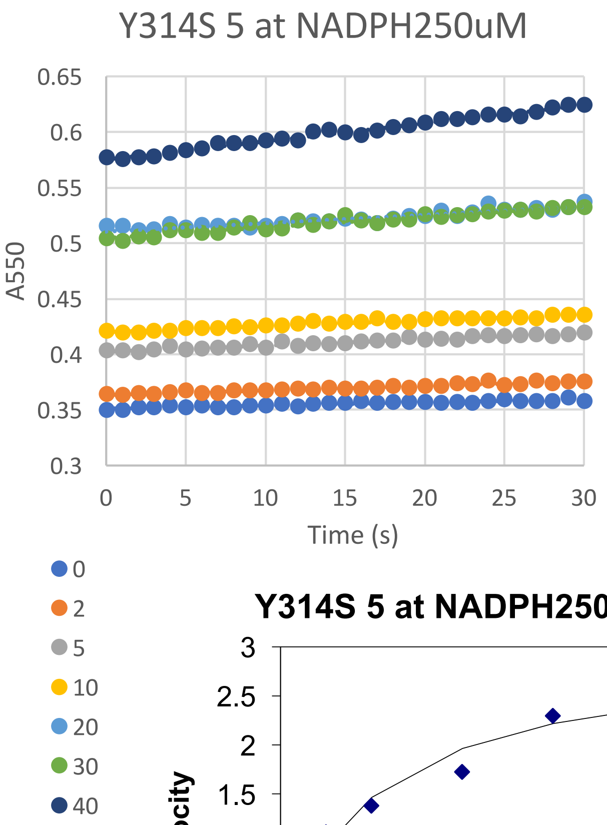

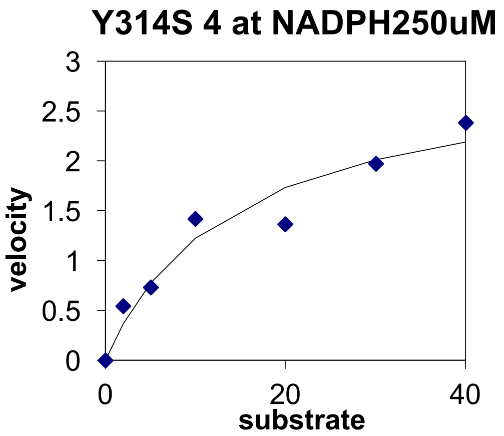

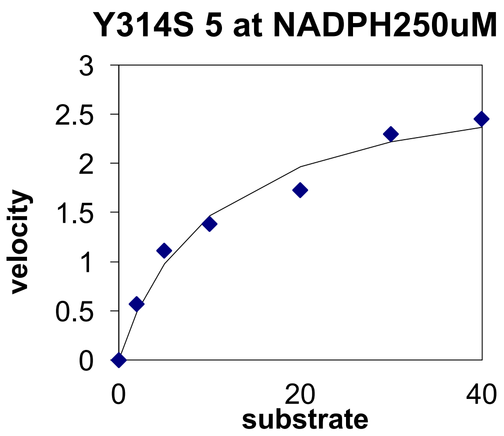

	1	2	3	4	5	Average	SD
Km (μM)=	14.62476831	9.402317583	17.56002178	14.13793672	10.16145187	13.17729925	3.019008771
Vmax (e/s)=	3.893403135	3.919003592	4.383900253	2.96273729	2.968351895	3.625479233	0.566424829

R235Q at 250 μM NADPH

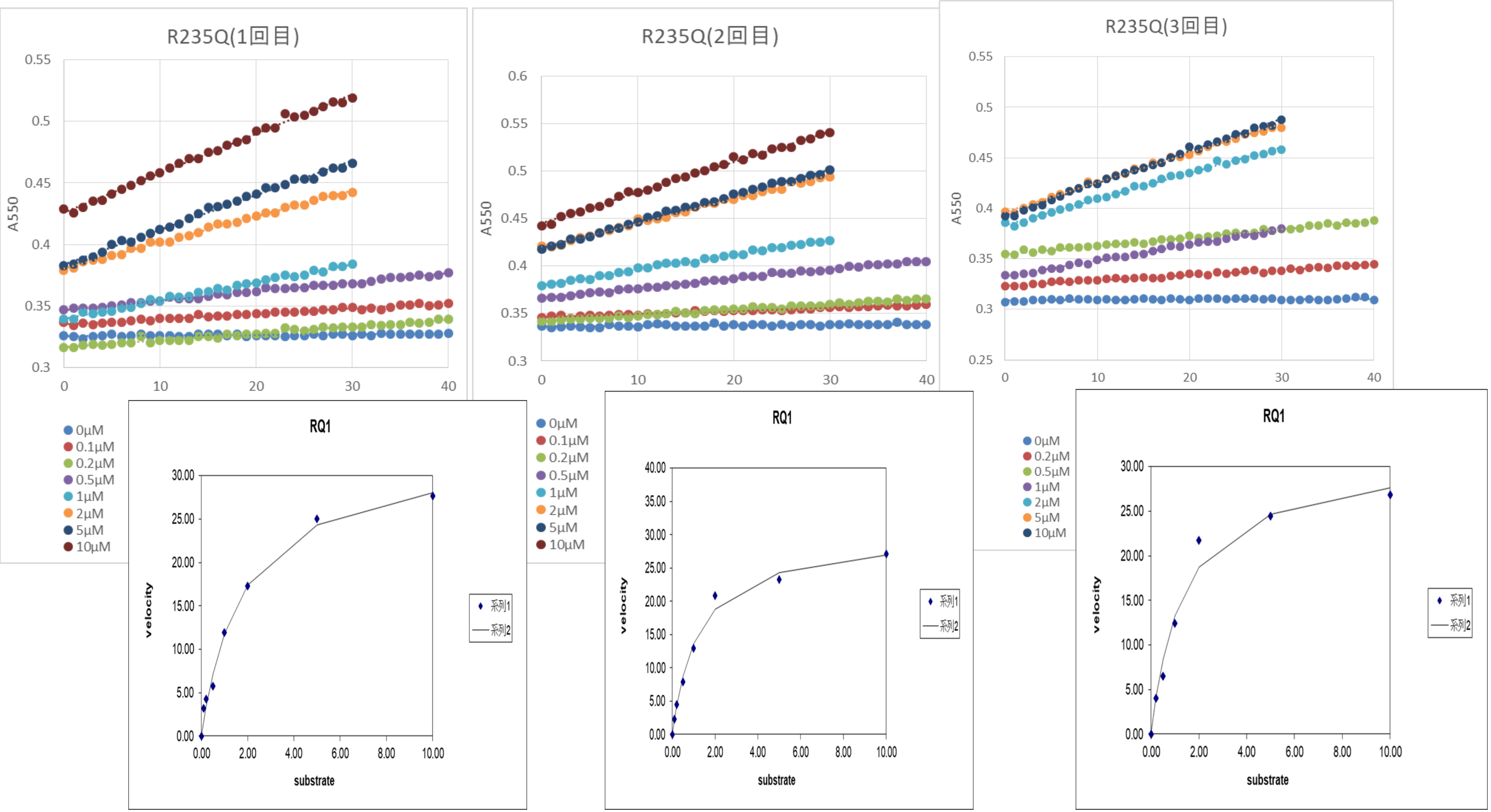

Km	1.8055	Vmax	33.1264

Km	1.226472	Vmax	30.29722

Km	1.359240	Vmax	31.413

	Km (μM)	Vmax (e/s)
R235Q	1.464±0.25	31.61±1.16

R244Q at 250 μM NADPH

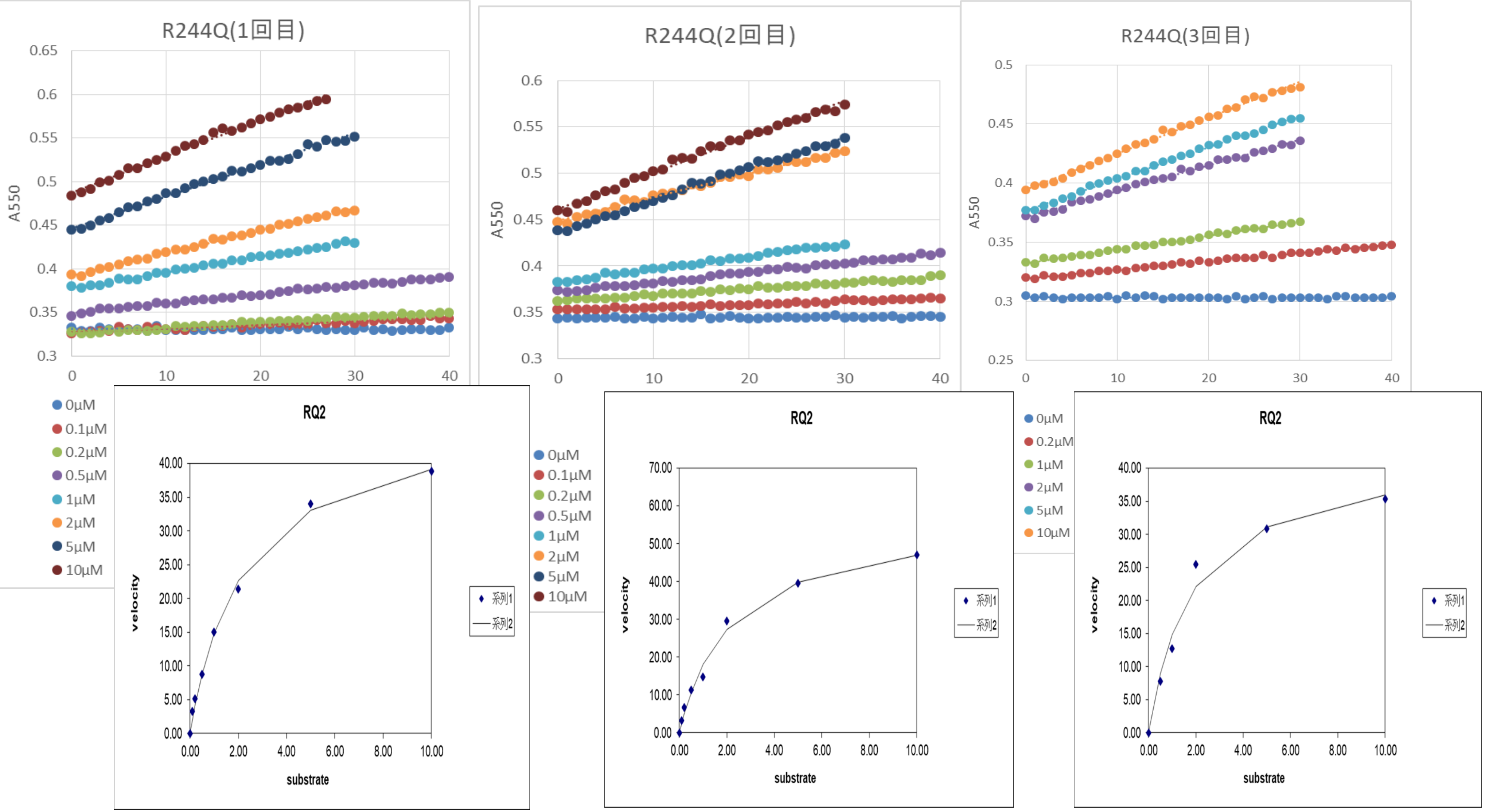

Km	2.2285103	Vmax	47.884027

Km	2.1746505	Vmax	57.252899

Km	1.8603524	Vmax	42.647381

	Km (μM)	Vmax (e/s)
R244Q	2.089±0.16	49.26±6.04

Cyt c reduction assay of wild-type FNR at 500 μM NADPH

numbers below X-axis: concentration of LFd (μM)

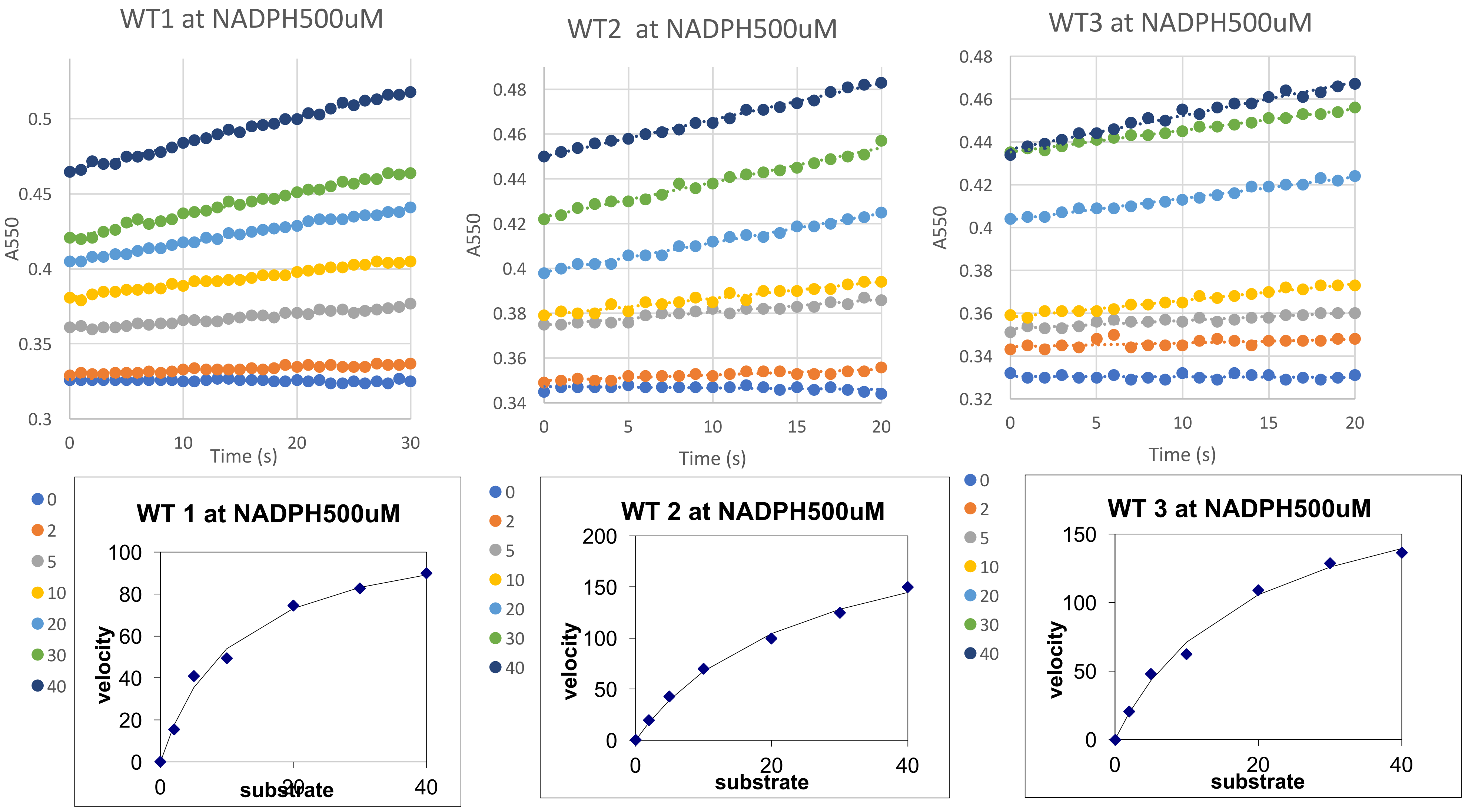

	1	2	3		Average	SD
Km (μM)=	24.74936223	18.9303608	23.3389757		22.33956624	2.478481906
Vmax (e/s)=	234.1982587	205.4605673	184.2837461		207.9808574	20.45529335

Y314S at 500 uM NADPH

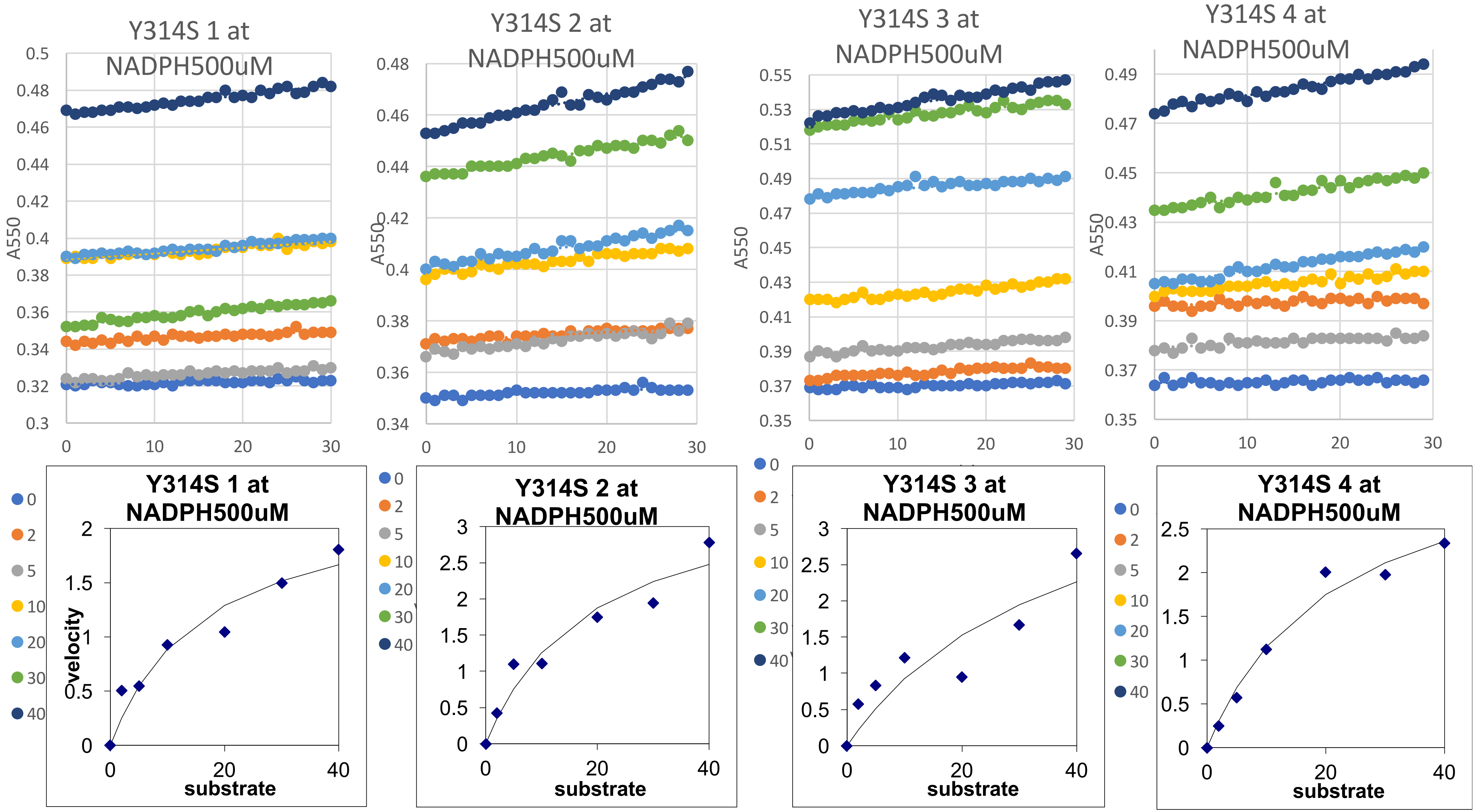

	1	2	3	4	Average		SD
Km (µM)=	16.62000622	19.13092072	15.60011307	21.27594863		18.15674716	2.2122603
Vmax (e/s)=	2.356690519	3.661335393	3.199938088	3.615057468		3.208255367	0.52345041

R235Q at 500 μM NADPH

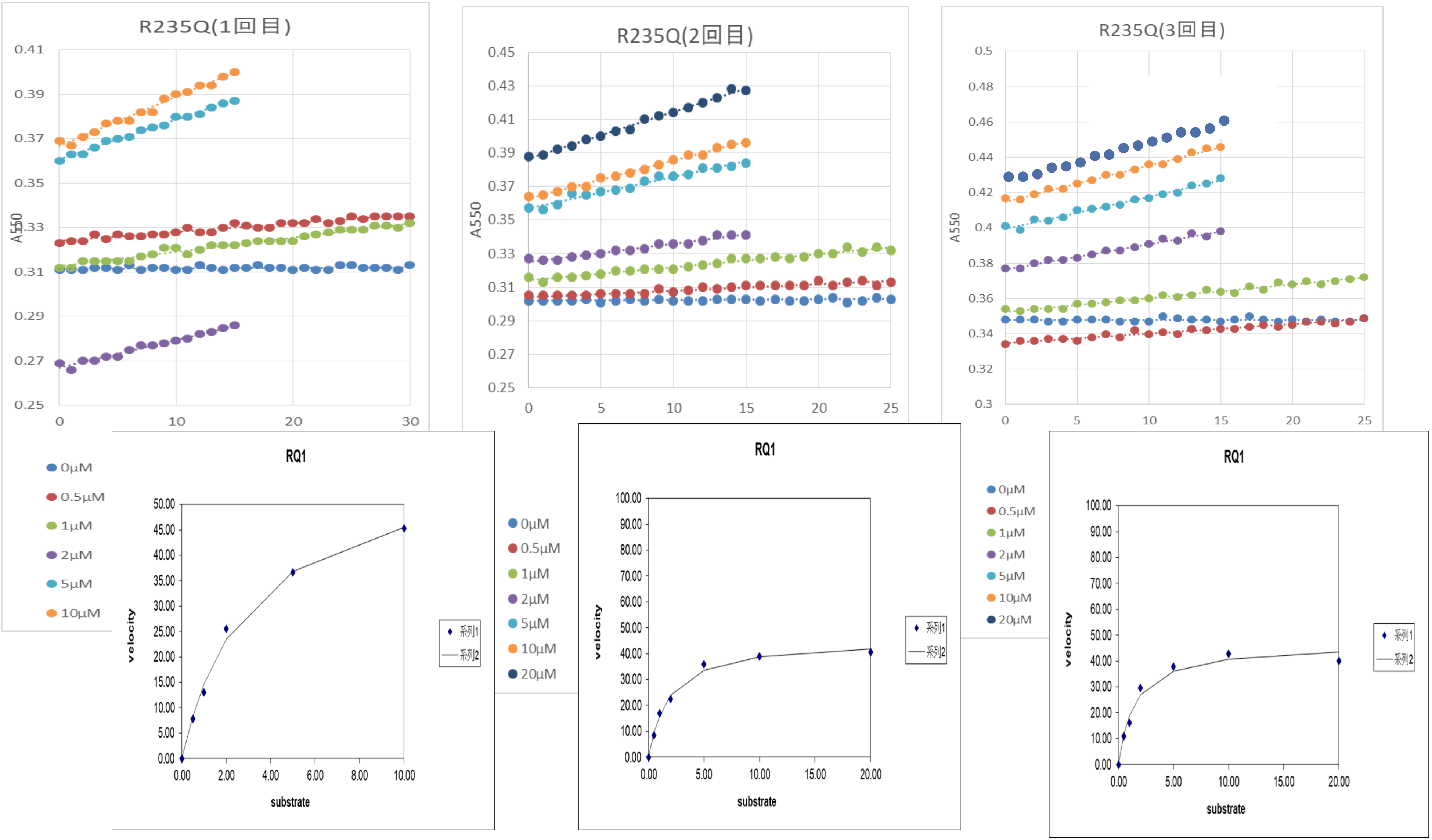

Km	3.045665	Vmax	59.35955

Km	1.838884	Vmax	45.81550

Km	1.430247	Vmax	46.47942

	Km (μM)		Vmax (e/s)	
R235Q	2.105±0.69		50.55±6.23	

R244Q at 500 μM NADPH

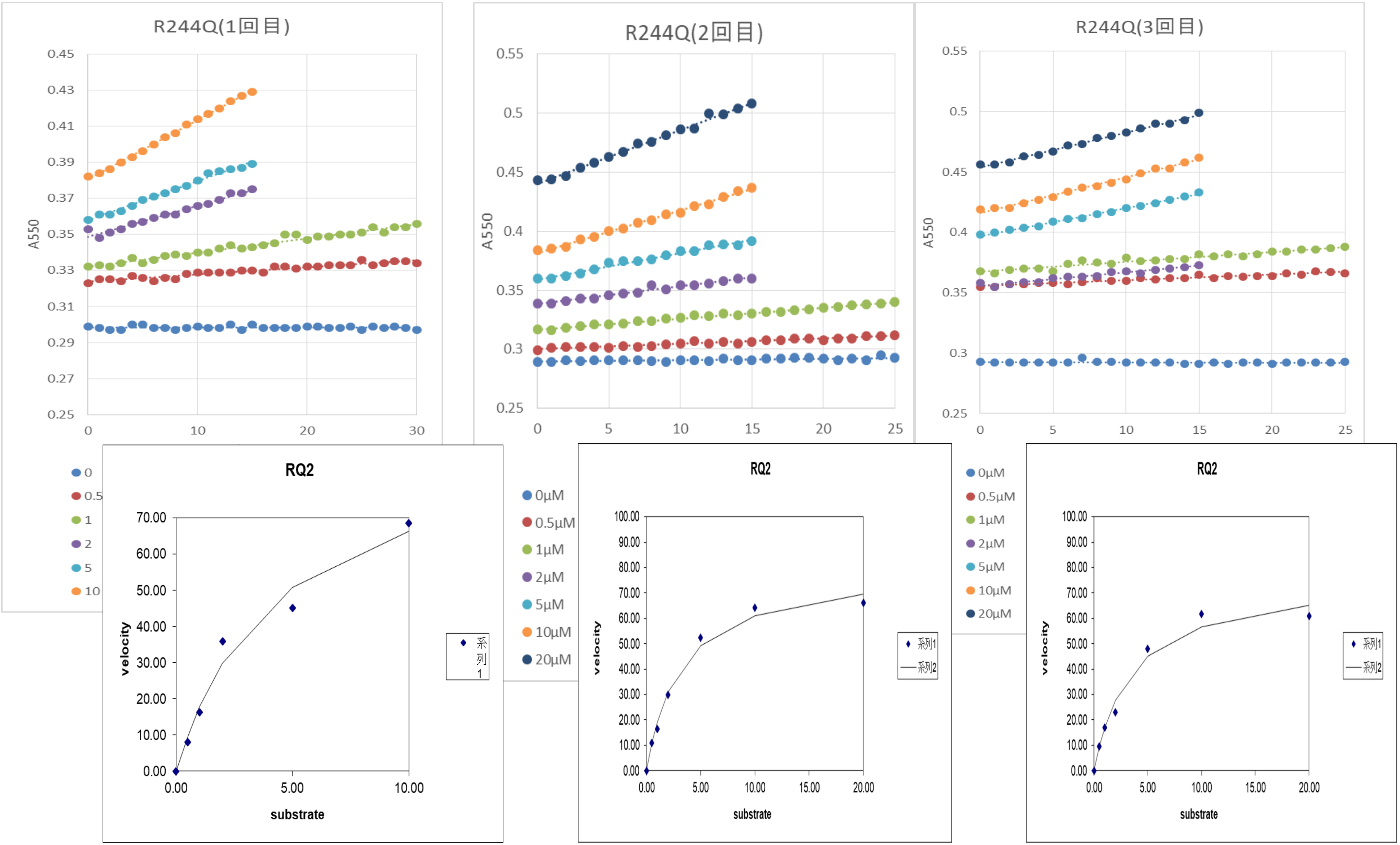

Km	4.400386	Vmax	95.640009

Km	3.14612	Vmax	80.3987

Km	3.4824	Vmax	76.4968

	Km (μM)		Vmax (e/s)	
R244Q	3.676±0.53		84.18±8.26	

Cyt c reduction assay of wild-type FNR at 1 mM NADPH

numbers below X-axis: concentration of LFd (μM)

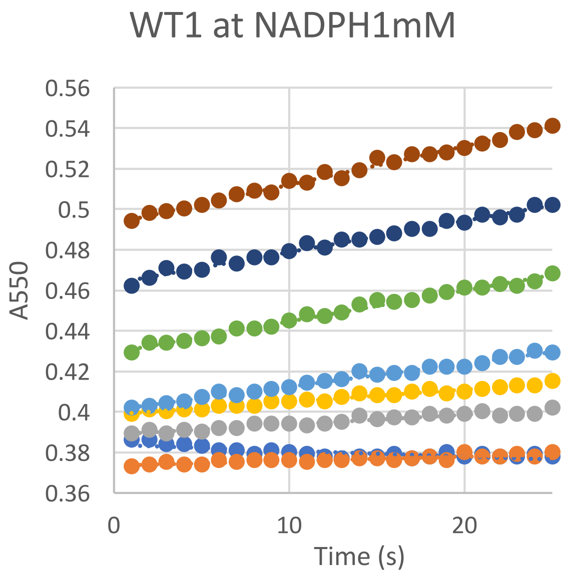

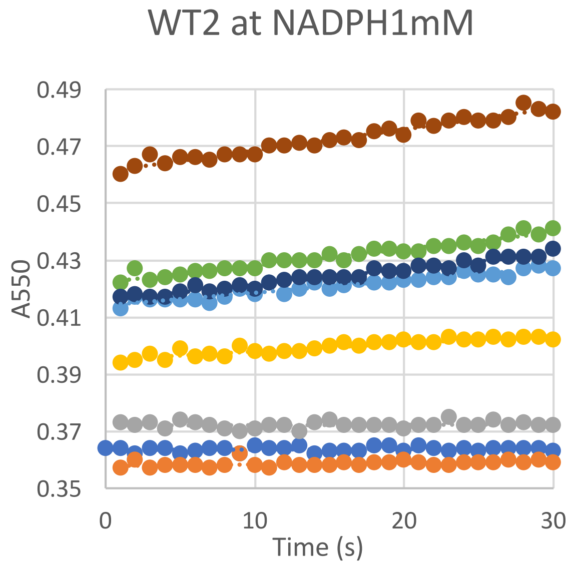

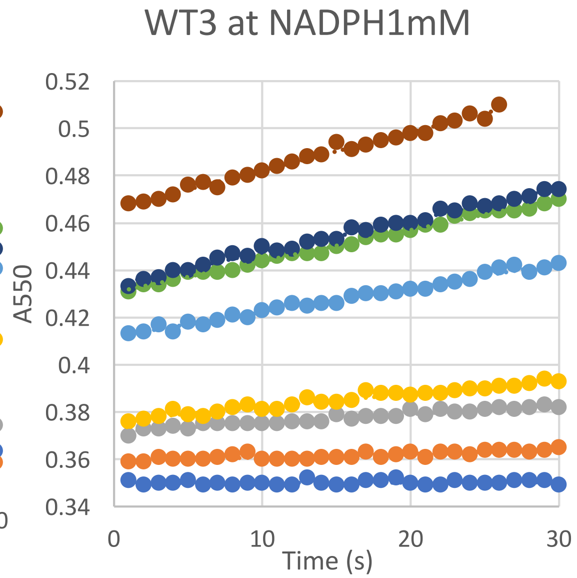

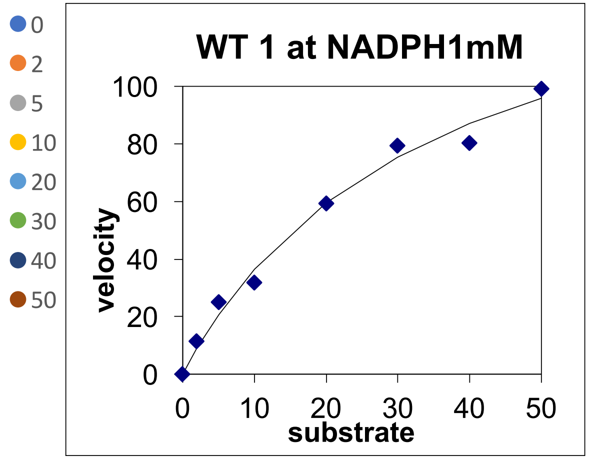

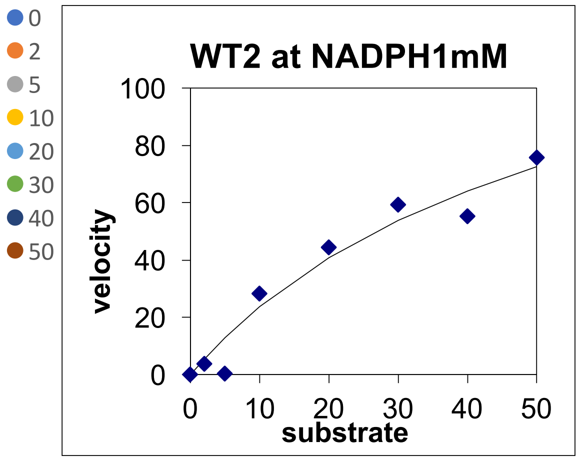

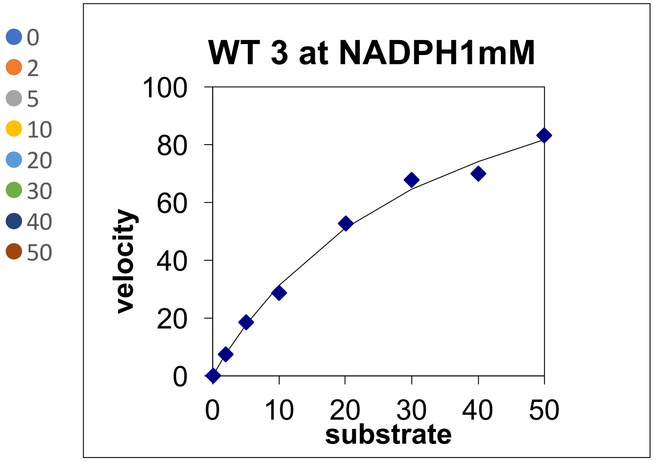

	1	2	3	4	Average	SD
Km (μM)=	34.27829	53.8883	33.434979	53.0924	43.673509	9.825431
Vmax (e/s)=	161.5780	150.7595	136.44268	167.0709	153.9628	11.6943

cont. wild-type FNR at 1 mM NADPH

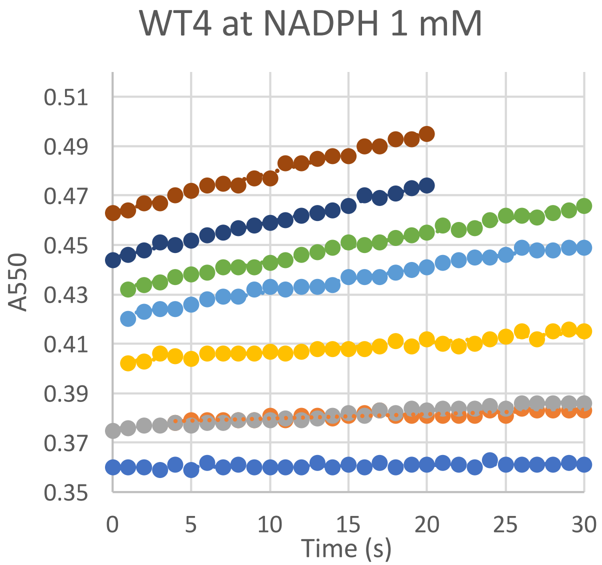

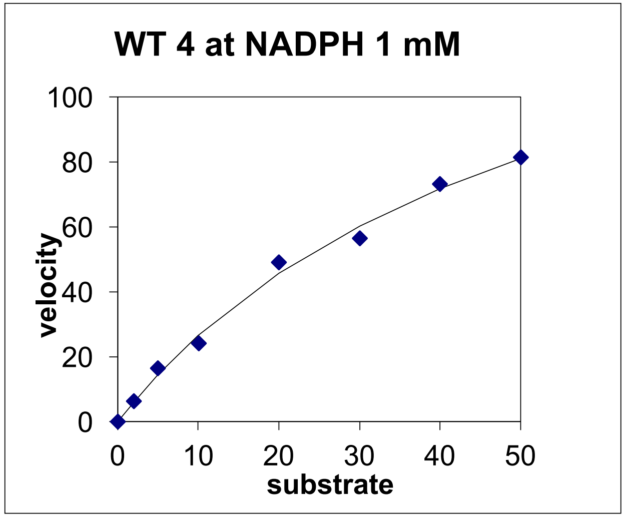

		1	2	3	4		Average	SD
Km (μM)=		34.27829	53.8883	33.434979	53.0924		43.673509	9.825431
Vmax (e/s)=		161.5780	150.7595	136.44268	167.0709		153.9628	11.6943

R235Q at 1 mM NADPH

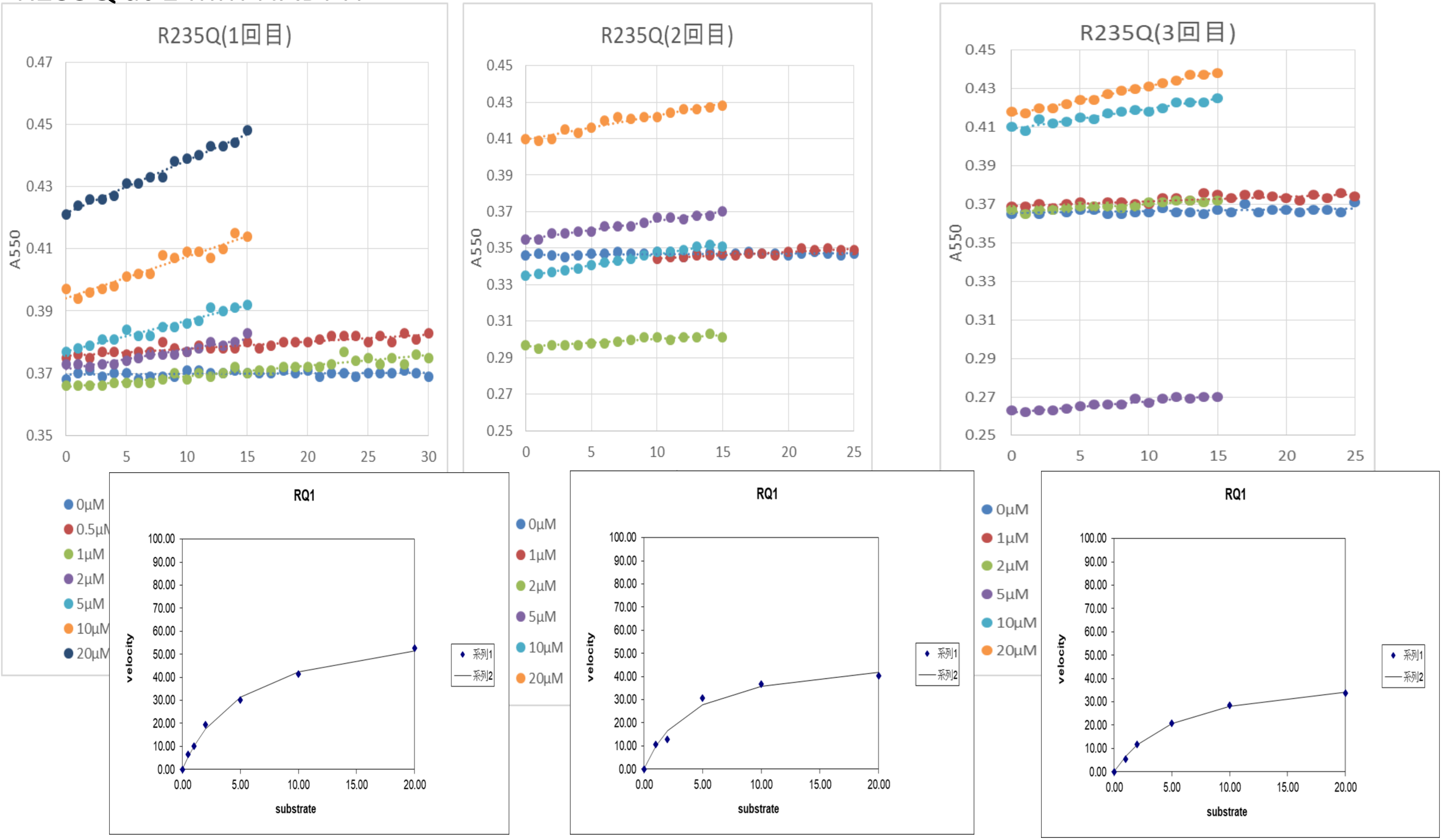

Km	5.42	Vmax	65.48

Km	4.1	Vmax	50.4

Km	5.61	Vmax	43.7

		R235Q	
Vmax (e/s)		53.19±9.11	
Km (μM)		5.04±0.67	

R244Q at 1 mM NADPH

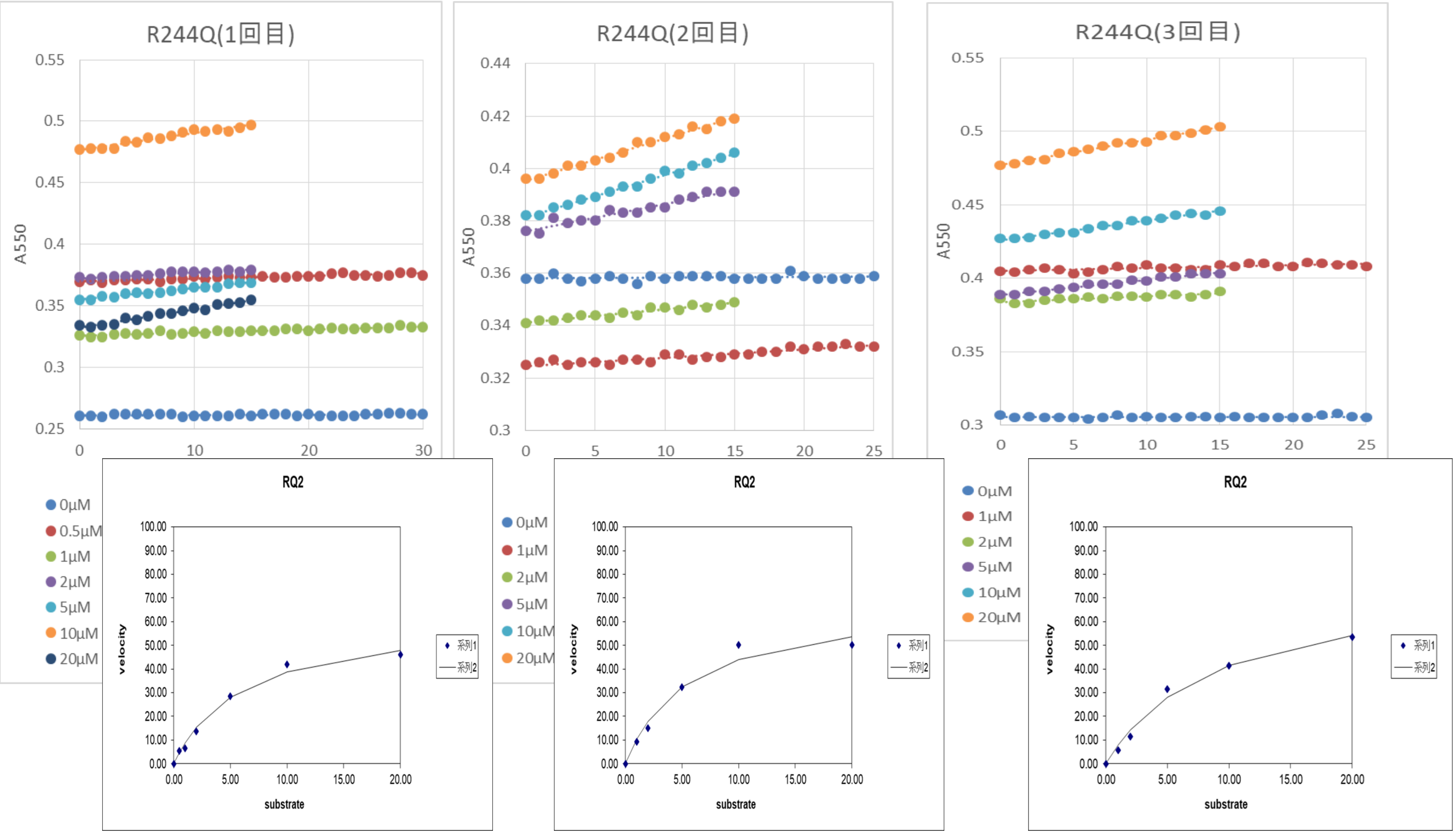

Km	6.19	Vmax	62.84

Km	5.62	Vmax	68.85

Km	8.84	Vmax	78.07

	R244Q	
Vmax (e/s)	69.92±6.26	
Km (µM)	6.88±1.4	

Cyt c reduction assay of Y314S FNR at 1 mM NADPH

numbers below X-axis: concentration of LFd (μM)

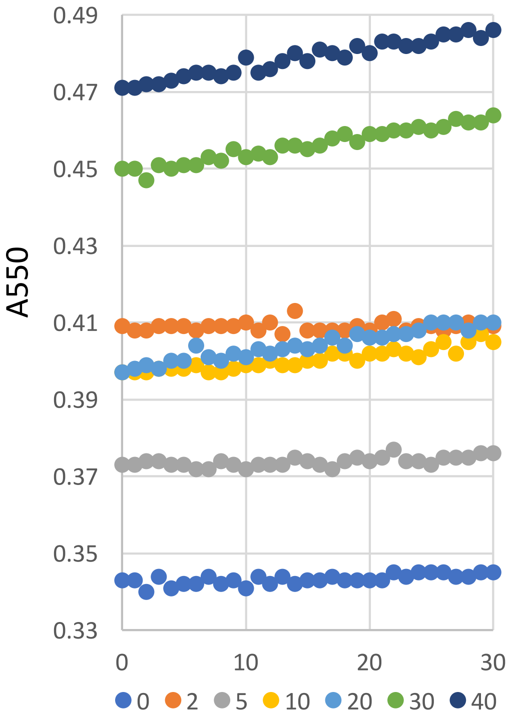

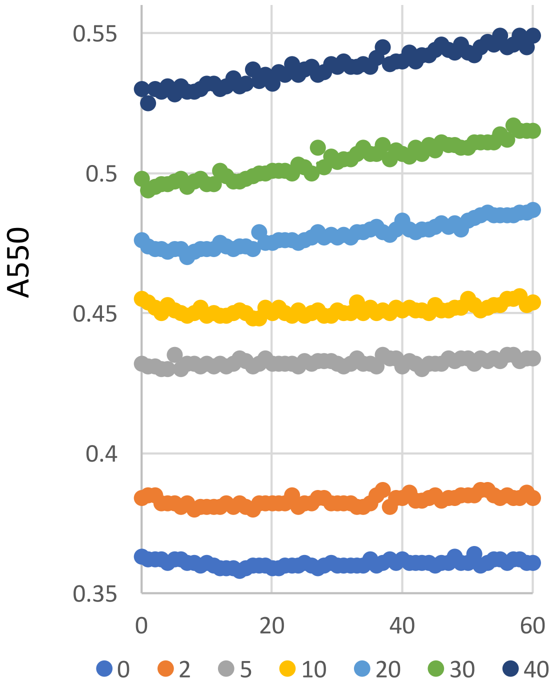

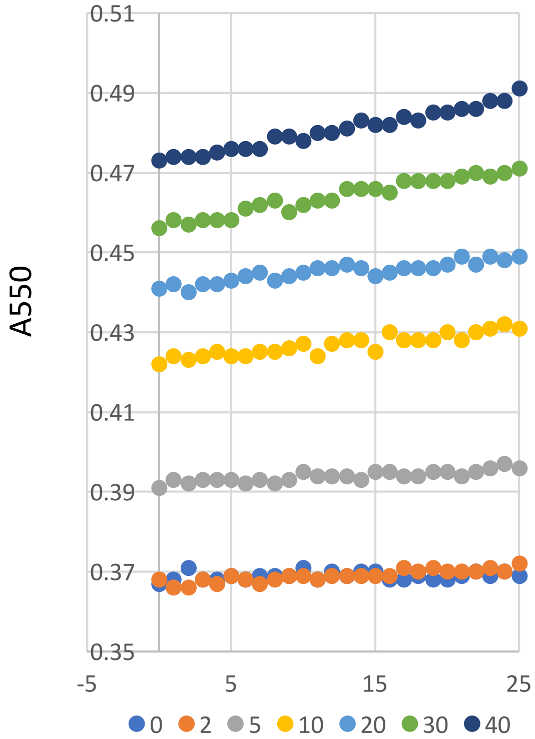

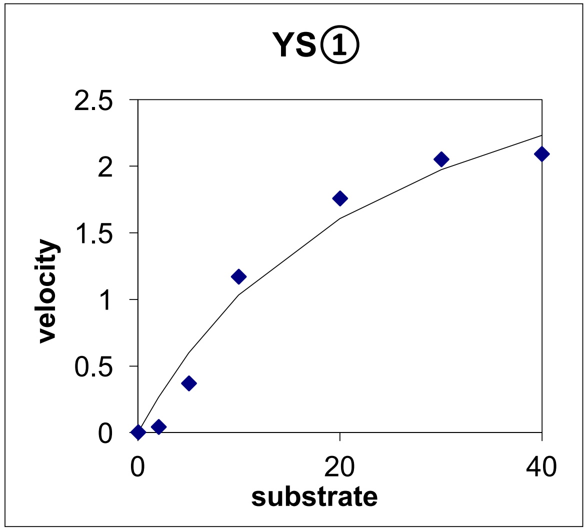

Km=	25.30715282
Vmax=	3.644712207

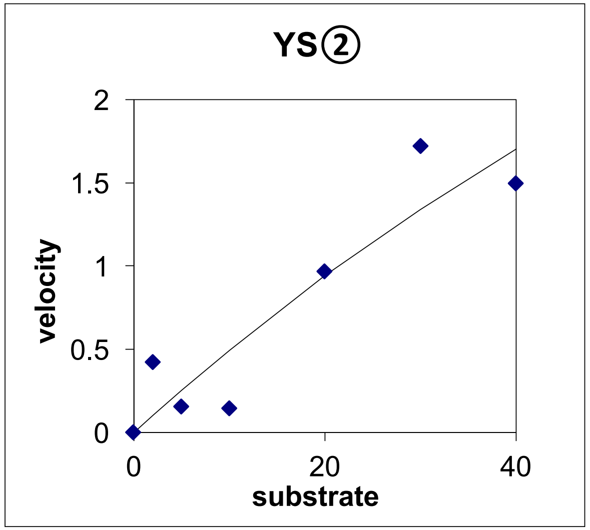

Not determined

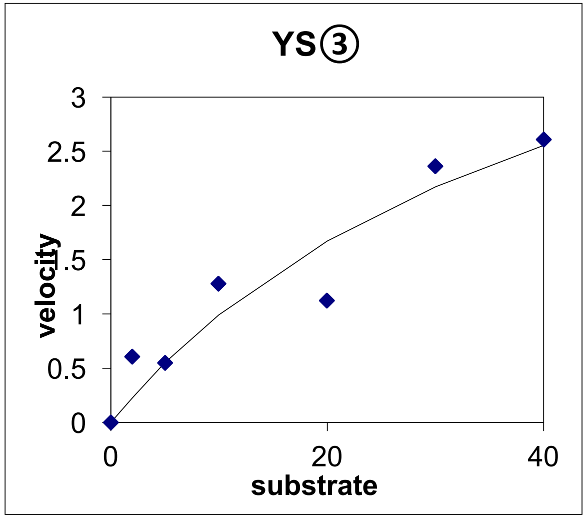

Not determined
