# Supplementary material for: NADP(H) allosterically regulates the interaction between ferredoxin and ferredoxin‐NADP+ reductase
Source: FEBS Open Bio. 2019 Nov 15;9(12):2126–36. doi: 10.1002/2211-5463.12752 (PMC6886308; doi:10.1002/2211-5463.12752)
Supplement: Supplementary file 3 — Fig S3. Kinetics of diaphorase reaction of wild‐type, mutant Y314, R235Q and R244Q FNRs. [file FEB4-9-2126-s003.pdf]

Diaphorase activity of wild-type FNR

X-axis: time(s) , Y-axis: A600nm, numbers below X-axis: concentration of NADPH(μM)

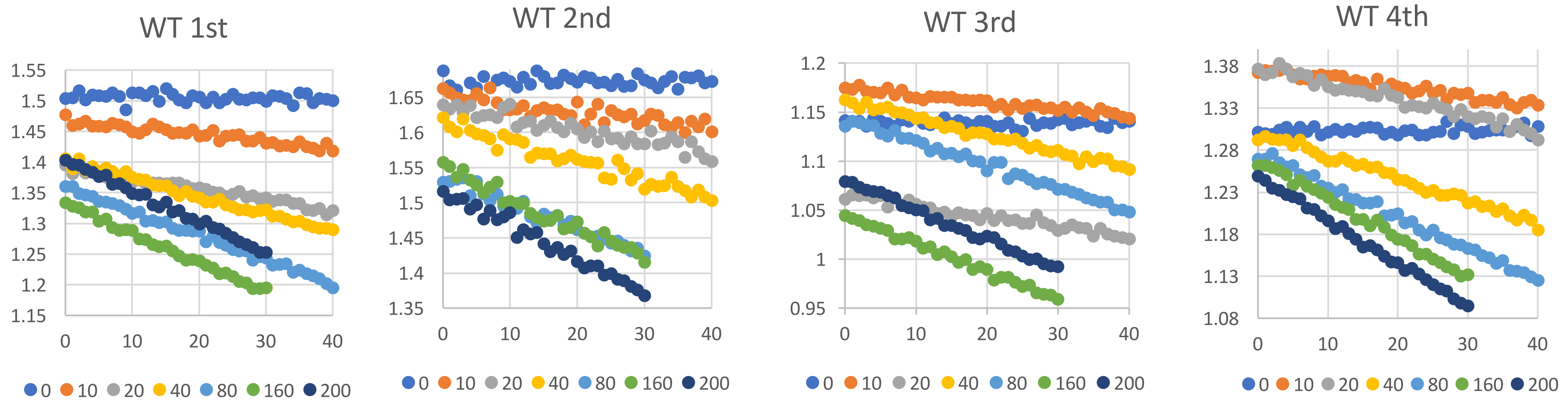

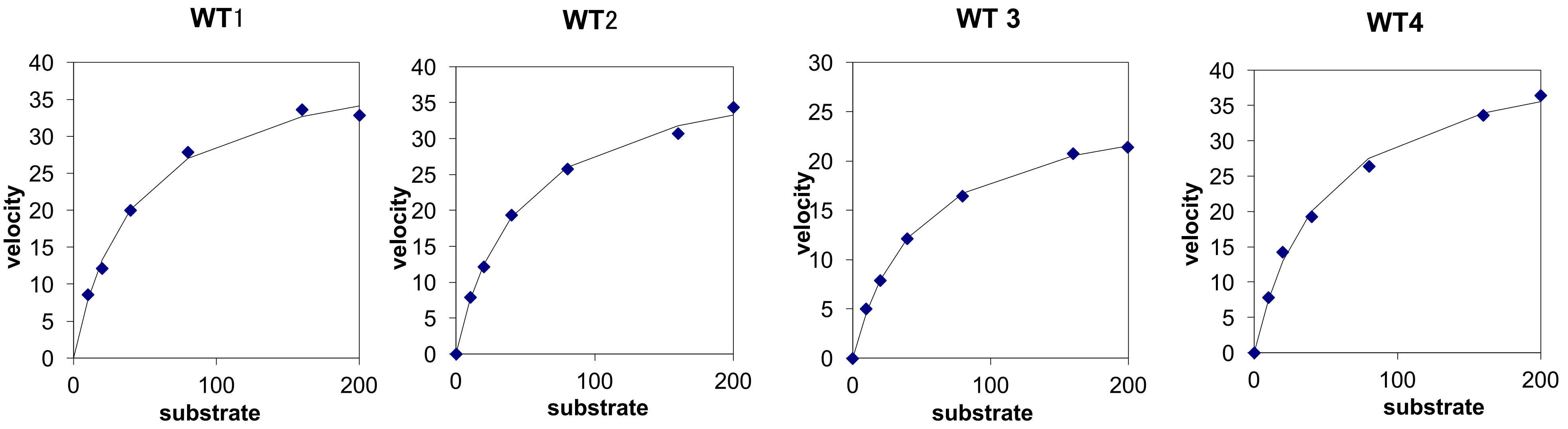

WT	1	2	3	4	Average	SD
Km (μM)	49.53	41.29	47.31	43.97	45.525	3.145612023
Vmax (2e/s)	44.95	38.81	26.62	42.1	38.12	6.985975236

k_{cat} (e^-/s) Ave 76 SD 14

Diaphorase activity of Y314S FNR

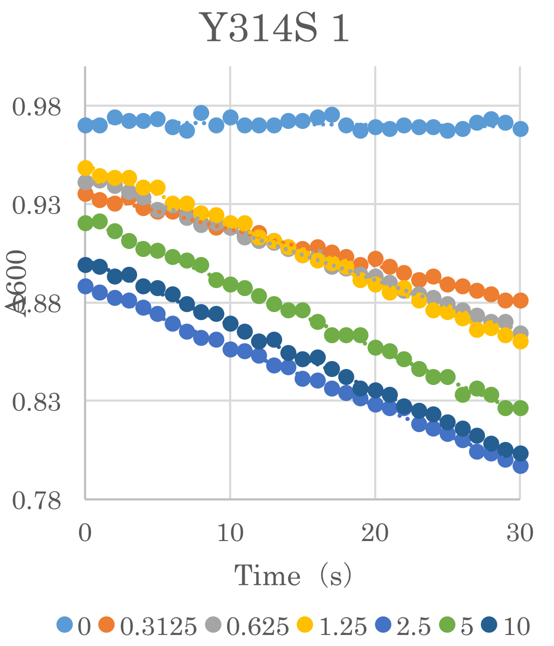

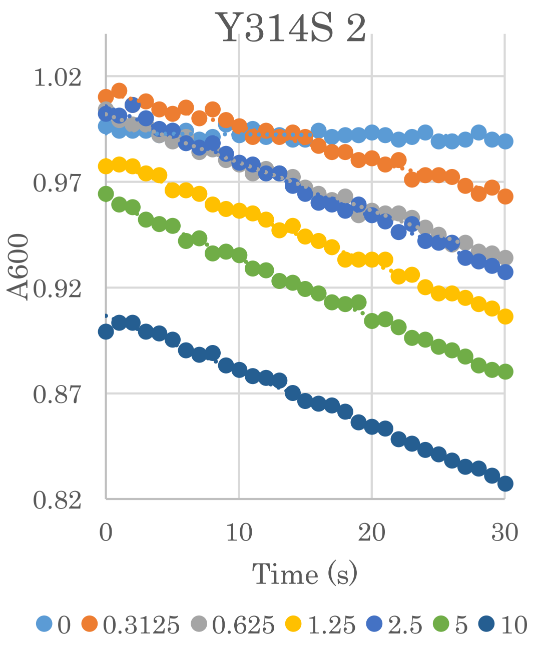

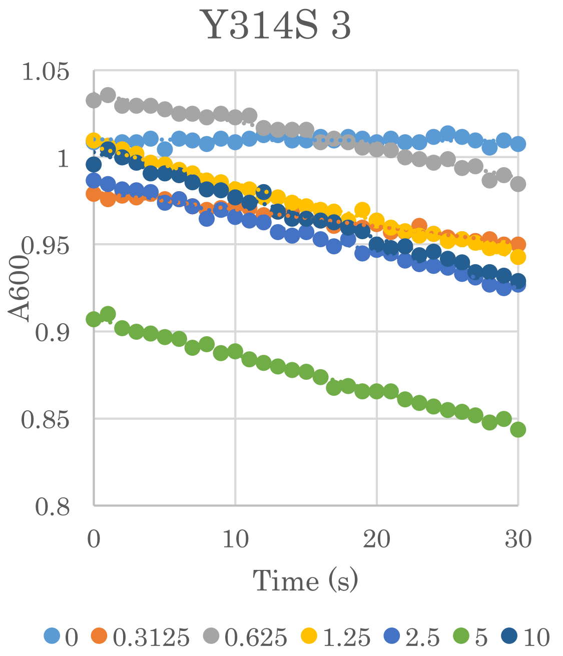

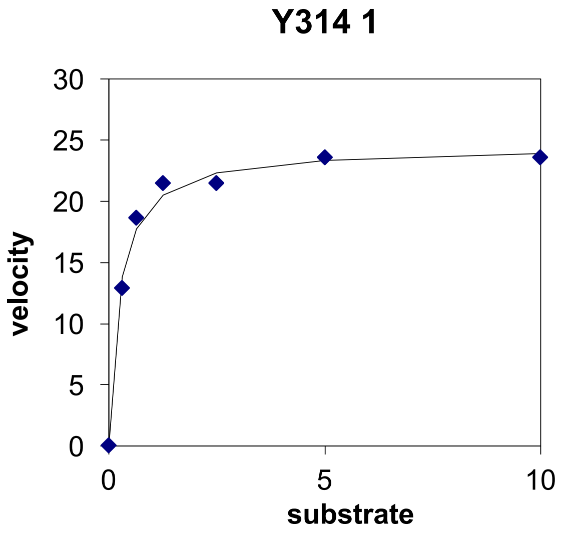

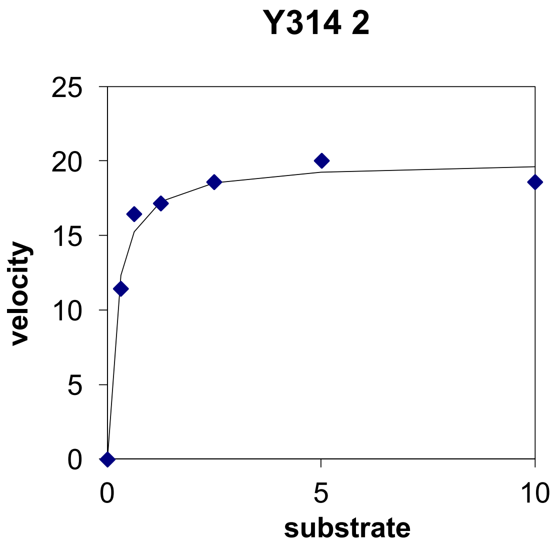

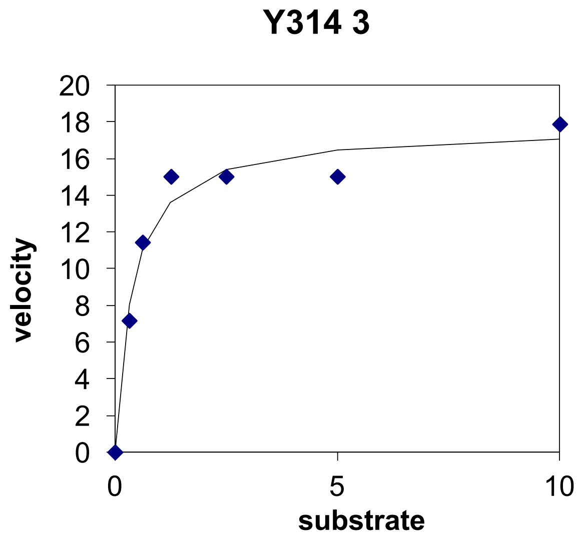

Y314S	1	2	3	Average	SD
Km (μM)=	0.195210554	0.240869156	0.373870879	0.26998353	0.075787479
Vmax (2e/s)=	20.00299715	24.47986808	17.69554103	20.72613542	2.81649547

k_{cat} (e^-/s) Ave 41 SD 6

Diaphorase activity of R235Q FNR

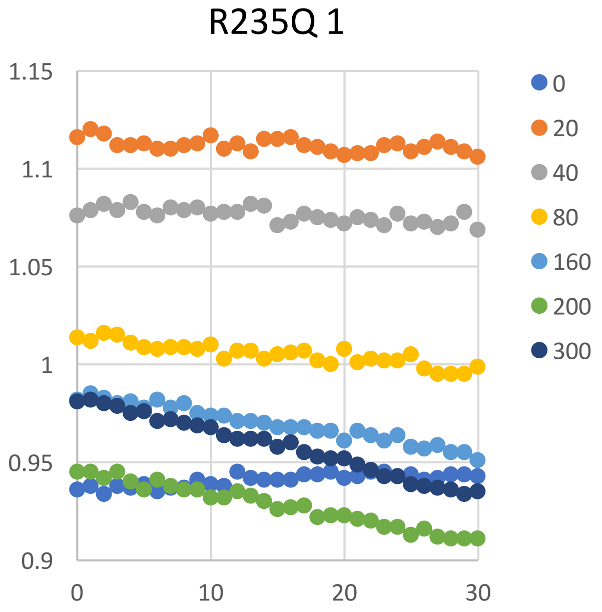

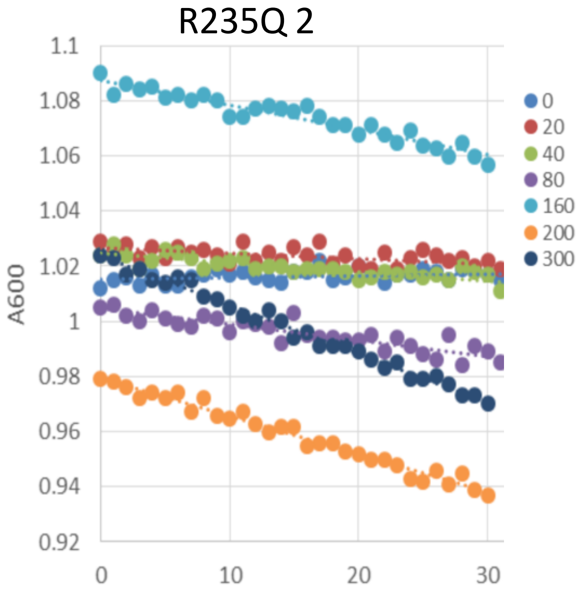

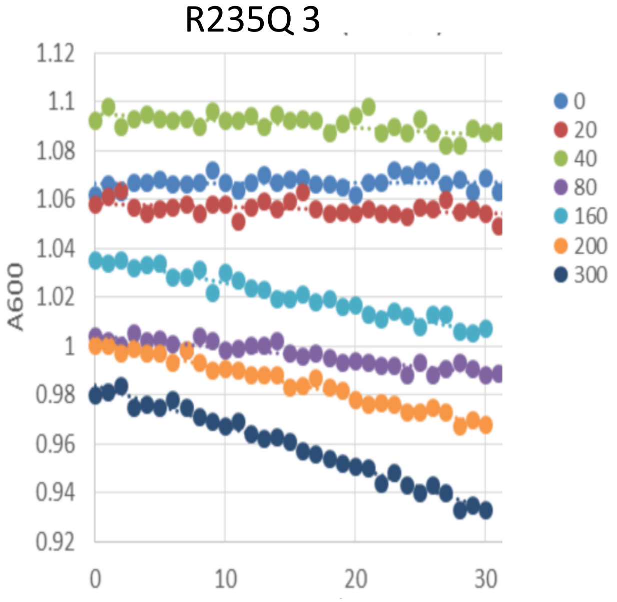

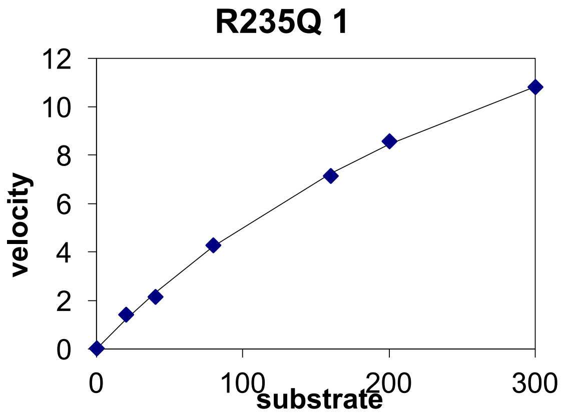

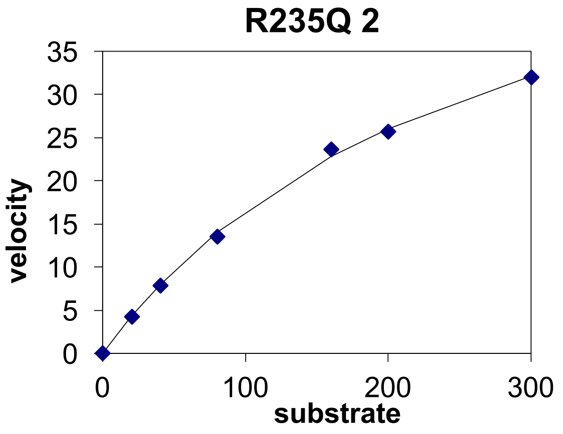

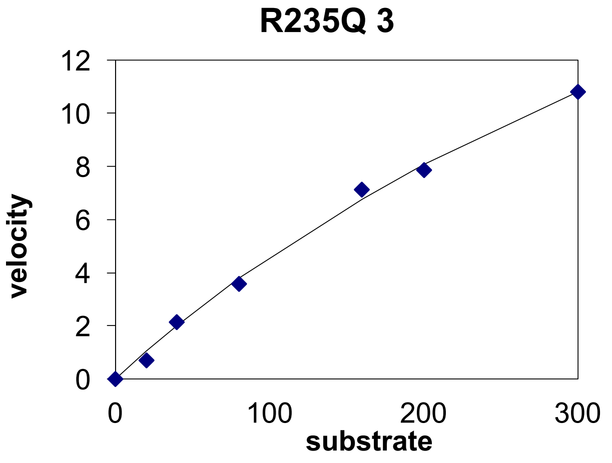

R235Q	1	2	3		Average	SD
Km (μM)	417	407	664		496	119
Vmax (2e/s)	26.2	28.2	34.9		29.8	3.7

k_{cat} (e^{-}/s) Ave 60 SD 7

Diaphorase activity of R244Q FNR

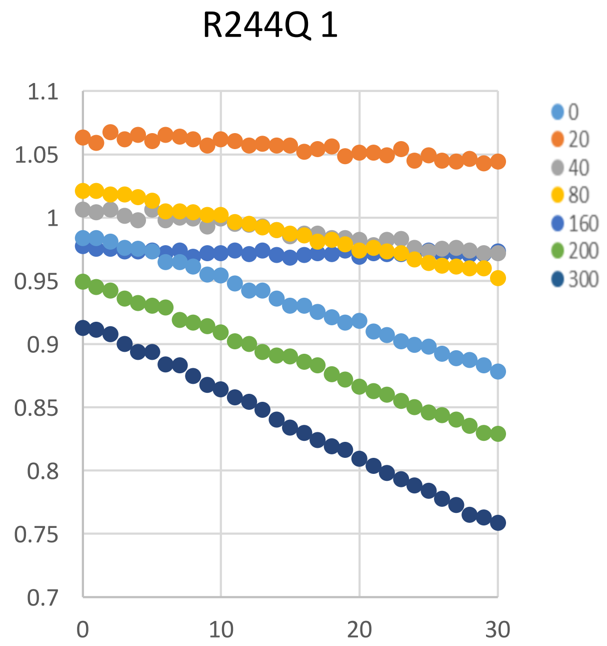

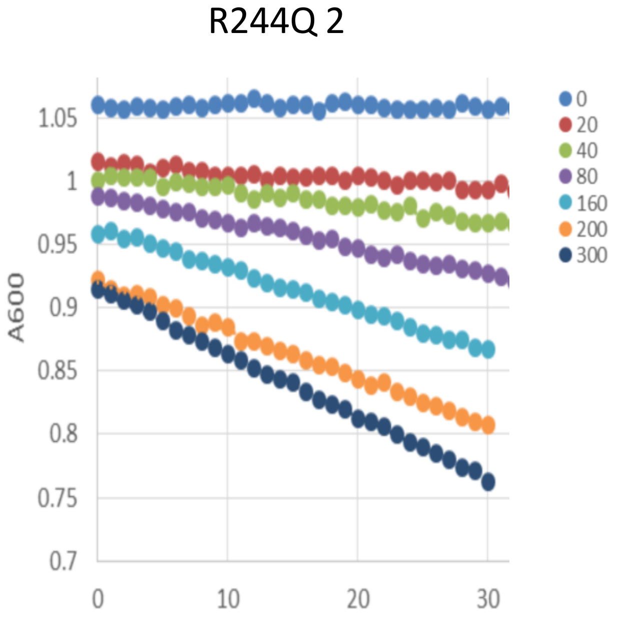

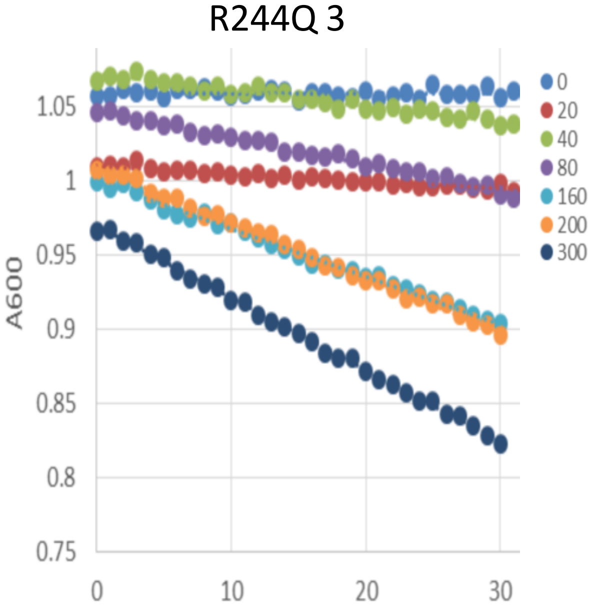

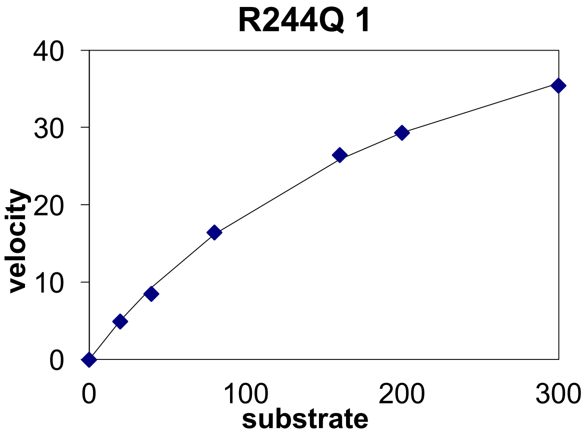

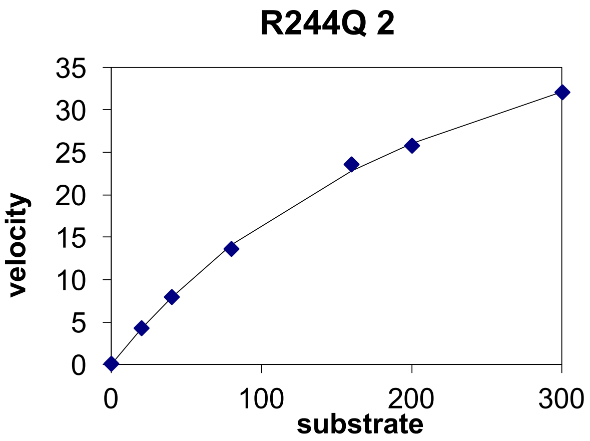

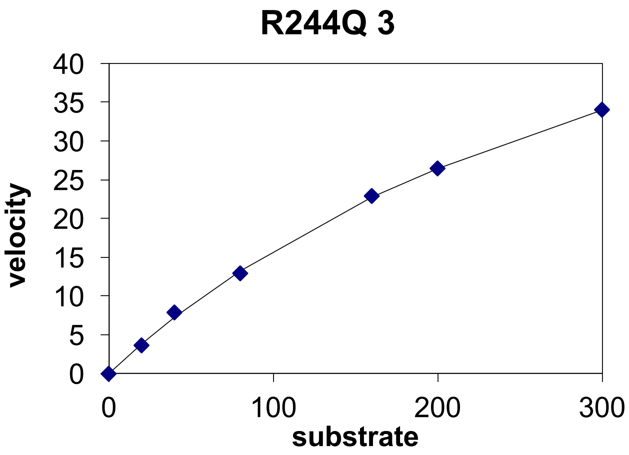

R244Q	1	2	3	Average	SD
Km (μM)	252	280	391	308	60
Vmax (2e/s)	67.1	62.8	78.2	69.4	6.5

k_{cat} (e^-/s) Ave 139 SD 13
