# Supplementary material for: NADP(H) allosterically regulates the interaction between ferredoxin and ferredoxin‐NADP+ reductase
Source: FEBS Open Bio. 2019 Nov 15;9(12):2126–36. doi: 10.1002/2211-5463.12752 (PMC6886308; doi:10.1002/2211-5463.12752)
Supplement: Supplementary file 4 — Table S1. Synthetic oligonucleotides used for the site‐directed mutagenesis. [file FEB4-9-2126-s004.pdf]

Table SI. Synthetic oligonucleotides used for the site-directed mutagenesis.

LFNR mutants		PCR primers (5'→3')
R235Q	FW[1]	5'-GACTACGCCGTCAGCCAGGAGCAGACG-3'
	RV[2]	5'-CGTCTGCTCCTGGCTGACGGCGTAGTC-3'
R244Q	FW	5'-GCGGCGGGGGAGCAGATGTACATCCAG-3'
	RV	5'-CTGGATGTACATCTGCTCCCCCGCCGC-3'
Y314S	FW	5′- GGAATGTGGAGGTCTCCTAACCGCTG -3′
	RV	5′- CAGCGGTTAGGAGACCTCCACATTCC -3′

[1]forward primers

[2]reverse primers
